# BcMFSR Is Required for Efficient Uptake of Exogenous dsRNA and Spray‐Induced Gene Silencing in *Botrytis cinerea*

**DOI:** 10.1111/mpp.70343

**Published:** 2026-09-20

**Authors:** Le Xu, Ze Yu, Ruonan Du, Huijie Ma, Xiaoxiao Hu, Ran Wei, Qiwang Hu, Cong Sheng, Wambui Doris Njoki, Pan Chen, Xuechao Shi, Cheng Tang, Huameng Zhang, Shaoxia Zhou, Haoming Zhong, Weishu Wang, Isashova Umida, Hongwei Zhao

**Affiliations:** ^1^ State Key Laboratory of Agricultural and Forestry Biosecurity, College of Plant Protection Nanjing Agricultural University Nanjing China; ^2^ Hunan Provincial Key Laboratory for Biology and Control of Plant Diseases and Insect Pests, College of Plant Protection Hunan Agricultural University Hunan China; ^3^ Department of Plant Protection Andijan Agricultural and Agrotechnology Institute Kuyganyor Uzbekistan

**Keywords:** gene silencing, grey mold, major facilitator superfamily (MFS), RNA interference (RNAi), RNA pesticide

## Abstract

Spray‐induced gene silencing (SIGS) uses exogenous double‐stranded RNA (dsRNA) to suppress pathogen virulence or growth‐related genes, offering a promising strategy for controlling grey mould caused by *Botrytis cinerea* at the gene level. In this study, we investigated the molecular mechanisms underlying the efficient uptake of dsRNAs by *B. cinerea* , making significant progress towards addressing key gaps in our understanding of SIGS. We identified four *B. cinerea* genes, *BcBMP1*, *BcSOD1*, *BcGAR2*, and *BcVELB*, as effective targets for grey mould control by employing an *Arabidopsis thaliana* SIGS screening system. Furthermore, by constructing a T‐DNA insertion mutant library, we identified the Δ*BcmfsR* mutant, which exhibited significantly impaired dsRNA internalization. Functional assays revealed that the *B. cinerea* Major Facilitator Superfamily protein, BcMFSR, is critical for pH‐dependent dsRNA uptake, facilitating SIGS efficacy. In the absence of functional BcMFSR, dsRNAs failed to reduce lesion size and *B. cinerea* virulence, regardless of the dsRNA sequence. BcMFSR is required for SIGS‐mediated silencing of four genes. Moreover, fluorescent labelling confirmed the localization of both BcMFSR and dsRNA in vesicular structures within hyphae, suggesting its involvement in endocytosis. Our discoveries not only established the novel role of BcMFSR in RNA uptake but also provided a foundation for improving the efficiency of RNA pesticides and broadening their agricultural applications.

## Graphical abstract

Spray‐induced gene silencing (SIGS) uses exogenous dsRNA to silence pathogen genes and control grey mould disease. We identify BcMFSR, an MFS protein required for dsRNA uptake and SIGS efficacy in *Botrytis cinerea*. Loss of BcMFSR abolishes dsRNA internalization and disease suppression, revealing a novel role for MFS proteins.

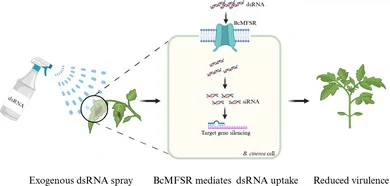

## 1 Introduction

RNA interference (RNAi) is a conserved gene‐silencing mechanism in which small RNAs (sRNAs) mediate sequence‐specific regulation of gene expression, often through the degradation or translational repression of target messenger RNAs (mRNAs). In plant–pathogen interactions, RNAi plays a pivotal role in regulating pathogen virulence and coordinating host immune responses (Chen and Rechavi 2022; Zhao and Guo 2022; Weiberg et al. 2013). Leveraging this natural regulatory mechanism, spray‐induced gene silencing (SIGS) has emerged as a promising RNAi‐based strategy that employs the exogenous application of sRNAs or double‐stranded RNAs (dsRNAs) to suppress pathogen gene expression (Koch et al. 2016; Wang and Jin 2017), Once the RNA molecules enter the host or pathogen, they inhibit pathogen growth or infection. This approach has been reported for controlling diseases caused by *Magnaporthe oryzae* (Chen et al. 2025), *Fusarium oxysporum* f. sp. *lycopersici* (Ouyang et al. 2023), *Verticillium dahliae* (Zhu et al. 2022) and *Botrytis cinerea* (Duanis‐Assaf et al. 2022). Unlike transgene‐dependent approaches, SIGS circumvents concerns associated with genetic modification while retaining high target specificity and environmental safety (Zhao et al. 2024; Luo et al. 2024).

*Botrytis cinerea* is a ubiquitous necrotrophic fungal pathogen that infects more than 2000 plant species and causes substantial economic losses worldwide (Li et al. 2018; Veloso and van Kan 2018). Exogenous application of dsRNAs targeting diverse fungal genes has been shown to suppress grey mould development; for example, applying dsRNA targeting the *B. cinerea* *DCL1/2* genes onto the surfaces of various vegetables, fruits, and ornamental plants markedly reduced lesion formation (Wang et al. 2016). Moreover, the application of dsRNAs targeting three key transcripts in the fungal ergosterol biosynthesis pathway (dsRNA‐erg) significantly reduced grey mould severity under in vitro conditions. In addition, combined treatment with dsRNA‐erg and ergosterol‐inhibiting fungicides achieved comparable protective efficacy while reducing the required fungicide dosage by up to 100‐fold (Duanis‐Assaf et al. 2022).

Despite these advances, how externally applied dsRNAs are internalized by *B. cinerea* cells and delivered to their intracellular targets remains largely unresolved (Qiao et al. 2021). Previous studies have shown that RNA trafficking between pathogens and hosts is frequently associated with vesicle transport and clathrin‐mediated endocytosis (CME). In *B. cinerea* , pathogen‐derived sRNAs can be delivered into plant cells via extracellular vesicles and internalized through CME (Cai et al. 2018; He et al. 2023), whereas in *Sclerotinia sclerotiorum* inhibition of CME significantly impairs dsRNA uptake (Wytinck et al. 2020). These observations suggest that endocytosis‐associated pathways play important roles in RNA transport. However, the molecular mechanisms underlying the uptake of exogenous dsRNAs by *B. cinerea* remain largely unknown. While endocytosis‐associated pathways have been implicated in RNA trafficking, whether they contribute to the uptake of externally applied dsRNAs in *B. cinerea* remains unresolved.

The Major Facilitator Superfamily (MFS) comprises one of the largest and most ubiquitous classes of membrane transporters across all domains of life (Reddy et al. 2012). Many MFS transporters mediate the transmembrane transport of various small molecules, including monosaccharides, polysaccharides, amino acids, peptides, drug compounds and nucleobases, playing crucial roles in diverse physiological processes (Marger and Saier 1993; Yan 2013; Wytinck et al. 2020). Although MFS transporters display remarkable functional versatility, whether they contribute to the uptake of extracellular RNA remains unknown. This knowledge gap is particularly striking given their established roles in diverse transmembrane transport processes.

Here, we identify a previously uncharacterized MFS protein in *B. cinerea* that is required for the uptake of extracellular dsRNA, which we designate as BcMFSR. Using a mutant library, we demonstrate that BcMFSR is essential for pH‐dependent endocytic uptake of dsRNA and for effective SIGS‐mediated suppression of fungal virulence. These findings reveal a previously unrecognized role for an MFS family protein in dsRNA uptake and provide new insights into the molecular mechanisms underlying SIGS in fungi.

## 2 Results

### 2.1 Exogenous dsRNA Effectively Inhibits *B. cinerea* Virulence via SIGS

**FIGURE 1:**
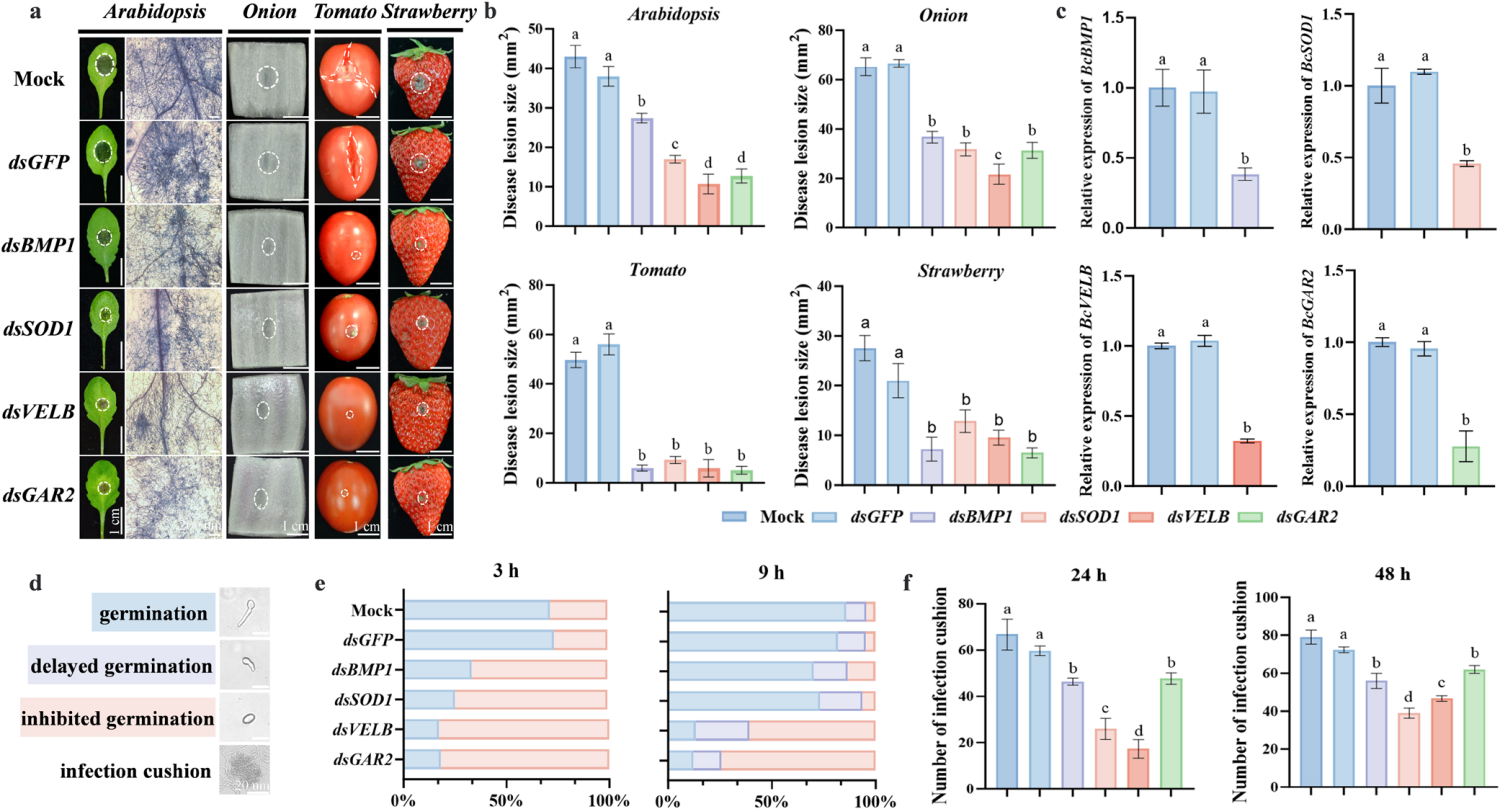
*dsBMP1*, *dsSOD1*, *dsVELB* and *dsGAR2* inhibit the growth and infection of *Botrytis cinerea*. (a) External application of water, *dsGFP*, *dsBMP1*, *dsSOD1*, *dsVELB* and *dsGAR2* to assess the pathogenicity of *B. cinerea* on *Arabidopsis thaliana* , tomato, onion and strawberry. (b) Lesion sizes were measured at 3 days post‐inoculation (dpi) in *A. thaliana* , onion, tomato and strawberry using ImageJ. (c) Reverse transcription‐quantitative PCR analysis of target gene expression in *B. cinerea* after exogenous application of four different dsRNAs. (d) Morphological classification and infection cushion formation of *B. cinerea* after exogenous application of four different dsRNAs. (e) Spore germination rates of *B. cinerea* after exogenous application of four different dsRNAs at 3 and 9 h. (f) Number of *B. cinerea* infection cushions after exogenous application of four different dsRNAs at 24 and 48 h. Significance was determined by ANOVA with Tukey's HSD test; different lowercase letters indicate significant difference (*p* < 0.05).

To assess whether dsRNAs targeting essential *B. cinerea* genes can suppress grey mould disease via SIGS, we selected four genes associated with virulence, growth and development. A 400‐bp sequence from the non‐conserved regions was selected from the transcript of *BcSOD1* (encoding Superoxide dismutase 1) (Rolke et al. 2004), *BcGAR2* (galacturonate reductase 2) (Zhang and van Kan 2013), *BcVELB* (velvet complex protein) (Yang et al. 2013), and *BcBMP1* (MAP kinase 1) (Zheng et al. 2000), targeted dsRNA was produced using *Escherichia coli* . Droplet application of *BcBMP1*‐dsRNA (*dsBMP1*), *BcSOD1*‐dsRNA (*dsSOD1*), *BcVELB*‐dsRNA (*dsVELB*) and *BcGAR2*‐dsRNA (*dsGAR2*) onto *Arabidopsis thaliana* leaves, tomato fruits and onion bulbs resulted in a marked reduction in necrotic lesions compared with the mock treatment (water) and the non‐targeting dsRNA control (*dsGFP*) (Figure 1a,b). Lesion sizes were significantly smaller in dsRNA‐treated plants compared to control treatments. Trypan blue staining at 48 h post‐inoculation (hpi) revealed that dsRNA application significantly reduced fungal colonization on *Arabidopsis* leaves (Figure 1a,b). Quantitative analysis showed that dsRNA treatment significantly reduced the expression of *BcBMP1*, *BcSOD1*, *BcVELB* and *BcGAR2* in infected tissues compared with *dsGFP* and mock treatments (Figure 1c), suggesting a direct correlation between the silencing of *B. cinerea* virulence genes and the observed reduction in disease severity. These findings suggest that *dsBMP1*, *dsSOD1*, *dsVELB* and *dsGAR2* are effective in silencing critical fungal genes, thereby limiting fungal growth and pathogenicity across multiple plant species.

We evaluated the effects of the four dsRNAs on *B. cinerea* germination and hyphal development by incubating spores with dsRNA and measuring germination, delayed germination and inhibited germination rates. After 3 h, *dsBMP1*, *dsSOD1*, *dsVELB* and *dsGAR2* treatments significantly reduced germination and increased delayed germination and inhibited germination compared with mock and *dsGFP* controls. No significant differences were observed between the mock and dsGFP treatments, indicating that non‐specific dsRNA had no effect on *B. cinerea* (Figure 1d,e); these inhibitory effects remained remarkably significant 9 h after co‐incubation. These results suggest that dsRNA treatments disrupt the early stages of fungal infection, likely by interfering with spore germination. At 12 h, extensive hyphal growth resulted in intertwined mycelia, making accurate observation and quantification of germination phenotypes difficult (Figure S1a). After that, we evaluated the formation of *B. cinerea* infection cushions, a critical step in pathogenesis, and found that it was significantly impaired by all four dsRNAs (Figure 1f and Figure S1a). We also recovered dsRNA from the slide and found that a substantial proportion remained intact throughout the experimental period, indicating that delayed germination was observed while extracellular dsRNA was still present and stable (Figure S1b). Taken together, this finding highlights the inhibition of SIGS across multiple fungal developmental processes, further supporting its potential as an effective antifungal agent.

To verify that the observed fluorescence originated from internalized rather than surface‐associated dsRNA, samples were treated with RNase A to degrade extracellular RNA prior to microscopy. Fluorescence microscope images captured 6 h after treatment revealed pronounced internalization of fluorescently labelled *dsBMP1* into *B. cinerea* spores and hyphae, confirming the successful uptake of the dsRNA (Figure S1c). Reverse transcription‐quantitative PCR (RT‐qPCR) analysis confirmed that *BcBMP1* expression was significantly reduced in *dsBMP1*‐treated hyphal samples, indicating effective gene silencing (Figure S1c,d). These results demonstrated the crucial role of *dsBMP1* in inhibiting *B. cinerea* by directly targeting a key virulence gene. Given its stable inhibitory effect and strong disease‐suppression activity, *dsBMP1* was selected to screen *B. cinerea* mutants for genes involved in *dsBMP1*‐mediated SIGS.

### 2.2 Screening of *B. cinerea* Genes Involved in SIGS Efficiency

**FIGURE 2:**
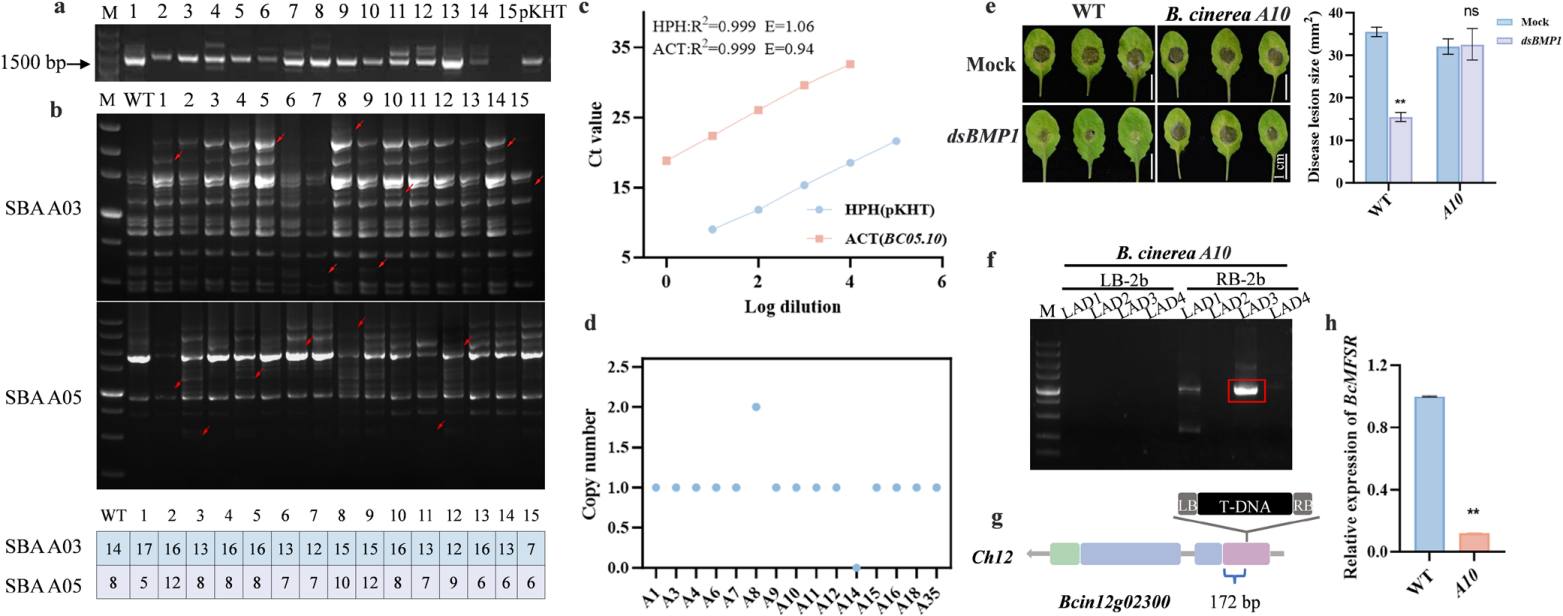
Validation and screening of a library of *Botrytis cinerea* T‐DNA insertion mutants. (a) PCR detection of the insertion of the *HPH* gene in the *B. cinerea* genome. (b) RAPD analysis was conducted on randomly selected transformants using primers SBA A03 and SBA A05. The number of bands for each sample is shown below. (c) Reverse transcription‐quantitative PCR (RT‐qPCR) was used to generate a standard curve for the *HPH* gene, with *B. cinerea* *Actin* serving as the internal reference. (d) Randomly selected transformants were tested for copy number using quantitative PCR, and the data were analysed using the Pfaffl method. (e) Pathogenicity assay and lesion size measurement of the *A10* transformant from the *B. cinerea* T‐DNA mutant library following application of *dsGFP* and *dsBMP1*. (f) HiTAIL‐PCR was used to identify the flanking sequences of the T‐DNA insertion site in the *A10* transformant. LB‐2b and RB‐2b represent left and right border nested primers, respectively, while LAD1–4 are four long degenerate primers. Potential target bands are circled in red. (g) Schematic representation of the T‐DNA insertion site in the *A10* transformant within the *B. cinerea* genome. Purple squares indicate the 5′ untranslated region (UTR), blue squares represent exons, grey lines depict introns, yellow squares indicate the 3′ UTR, and black squares represent T‐DNA. (h) RT‐qPCR analysis of *BcMFSR* expression in WT and *A10* transformants. Significance was determined using Student's *t*‐test: **p < 0.01; ns, not significant.

To identify critical *B. cinerea* genes that may affect SIGS, we constructed a *B. cinerea* mutant library using the *Agroacterium tumefaciens*‐mediated transformation (ATMT) method (Schumacher et al. 2014). A total of 3000 transformants were generated using a hygromycin resistance cassette for selection. The library was thoroughly characterized before being employed in subsequent screening experiments. To assess T‐DNA insertion coverage and randomness, we amplified the hygromycin phosphotransferase (*HPH*) gene and performed random amplified polymorphic DNA (RAPD) analysis on 15 randomly selected transformants. Results showed that approximately 93.3% of the transformants had the resistance gene inserted and exhibited distinct insertion patterns, indicating the mutant library displays high insertional diversity and randomness (Figure 2a,b). Furthermore, qPCR analysis was performed to assess the copy number of the T‐DNA insertions in the library (Song et al. 2002). Based on a standard curve generated for the single‐copy *HPH* gene (Figure 2c), we found that 86% of the transformants contained a single copy of the T‐DNA insertion (Figure 2d and Figure S2a), indicating that the library is of high quality and suitable for genetic screening. A workflow diagram illustrating the generation of the mutant library, its quality assessment, and the identification of candidate mutants is presented in Figure S2b.

Following the characterization of the ATMT library, a screening was carried out to identify mutants showing reduced sensitivity to *dsBMP1* treatment, based on a set of predefined criteria. The screening criteria were as follows: [1] Wild‐type (WT) *B. cinerea* should cause a more severe disease phenotype on *Arabidopsis* leaves treated with *dsGFP* than on leaves treated with *dsBMP1*, ensuring that the SIGS screening system is functioning properly (*dsGFP*@ *B. cinerea* ^WT^ > *dsBMP1*@ *B. cinerea* ^WT^). [2] *Arabidopsis* leaves treated with *dsGFP*, regardless of whether they were infected with WT strain or *B. cinerea* mutants, should display similar disease severity, ensuring that the T‐DNA insertion does not cause disease susceptibility (*dsGFP*@ *B. cinerea* ^WT^ = *dsGFP*@ *B. cinerea* ^Mu^). [3] *Arabidopsis* leaves treated with *dsGFP* and infected with *B. cinerea* mutant should show disease phenotypes similar to those treated with *dsBMP1*, confirming that *B. cinerea* mutants affect the SIGS response mediated by *dsBMP1* (*dsGFP*@ *B. cinerea* ^Mu^ = *dsBMP1*@ *B. cinerea* ^Mu^).

Following the screening of about 200 transformants from the mutant library, the *B. cinerea A10* mutant was identified, which exhibited a significant defect in its response to *dsBMP1*‐mediated SIGS. *Arabidopsis* leaves infected with the *B. cinerea* *A10* mutant and treated with *dsGFP* exhibited a comparable level of necrosis to that of leaves infected with the WT strain and treated with *dsGFP* (Figure 2e), indicating that the *B. cinerea A10* mutant retained full virulence. However, when leaves were infected with the *B. cinerea A10* mutant and treated with *dsBMP1*, they failed to show the expected reduction in necrosis observed in wild‐type (WT)‐infected leaves, indicating a defect in the SIGS pathway. HiTAIL‐PCR results revealed that the T‐DNA was inserted into the 5′ untranslated region (UTR) of *Bcin12g02300*, which encodes a major facilitator superfamily (MFS) transporter in *B. cinerea* (Figure 2f,g). RT‐qPCR analysis confirmed that the mRNA expression level of *BcMFSR* was significantly reduced in the *B. cinerea A10* mutant compared to the WT (Figure 2h).

During the screening of the mutant library, we also obtained some other results. For example, one insertion occurred in the 5′ UTR of *Bcin09g05170*, encoding an AB hydrolase domain protein involved in lipid droplet biogenesis, and another in the promoter region of *Bcin14g01900*, encoding a SAC1‐like phosphatidylinositol phosphatase associated with secretory membrane trafficking (Figure S3) (Liu and Bankaitis 2010). Previous studies also identified 21 predicted *B. cinerea* target genes of *A. thaliana* ‐derived sRNAs, many of which are related to vesicular transport (Cai et al. 2018). However, because *BcMFSR* showed the strongest and most reproducible phenotype in subsequent validation assays, it was selected for further functional characterization in this study. Although their roles in SIGS remain unclear, the identification of these candidates suggests that vesicle‐associated processes may influence SIGS in *B. cinerea* .

### 2.3 Characterization of *BcMFSR* in Growth, Morphology and Pathogenicity

**FIGURE 3:**
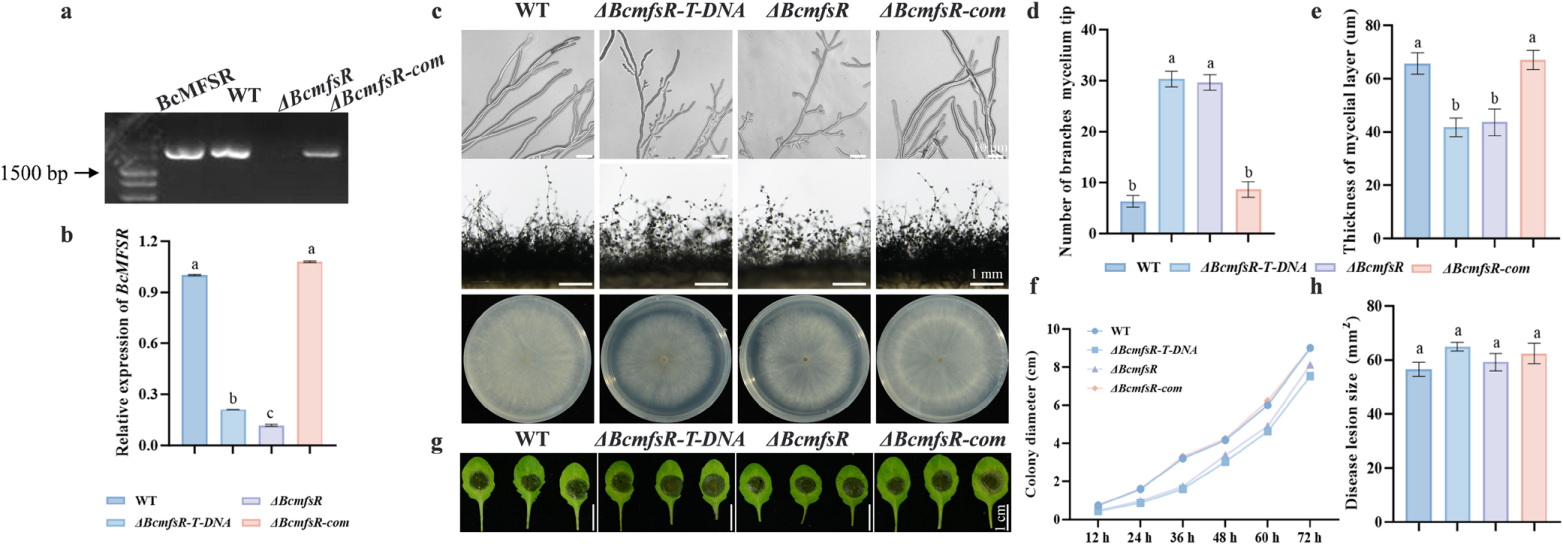
BcMFSR mutation contributes to an increase in mycelium branching, reduced layer depth, and the colony growth rate. (a) PCR verification of the construction of Δ*BcmfsR* and Δ*BcmfsR‐com* strains. (b) Reverse transcription‐quantitative PCR analysis of *BcMFSR* expression in wild type (WT), Δ*BcmfsR‐T‐DNA*, Δ*BcmfsR*, and Δ*BcmfsR‐com* strains. (c) Hyphal tip morphology, hyphal layer thickness, and colony growth rate of WT, Δ*BcmfsR‐T‐DNA*, Δ*BcmfsR*, and Δ*BcmfsR‐com* strains. (d, e) Quantification of the number of hyphal tip branches (d) and hyphal layer thickness (e) in WT, Δ*BcmfsR‐T‐DNA*, Δ*BcmfsR*, and Δ*BcmfsR‐com* strains. (f) Quantification of colony growth rates of WT, Δ*BcmfsR‐T‐DNA*, Δ*BcmfsR*, and Δ*BcmfsR‐com* strains. (g) Pathogenicity analysis of WT, Δ*BcmfsR‐T‐DNA*, Δ*BcmfsR*, and Δ*BcmfsR‐com* on *Arabidopsis thaliana* . (h) Quantification of lesion areas caused by WT, Δ*BcmfsR‐T‐DNA*, Δ*BcmfsR*, and Δ*BcmfsR‐com* on *A. thaliana* . Statistical significance was determined by ANOVA with Tukey's HSD test; different lowercase letters indicate significant difference (*p* < 0.05).

To investigate the role of *BcMFSR* in the RNA‐mediated gene silencing pathway of *B. cinerea* , we generated a series of mutants, including a knockout strain (Δ*BcmfsR*) and its complemented strain (Δ*BcmfsR‐com*), in which the *BcMFSR* gene was reintroduced under the control of its native promoter (Figure 3a,b). The role of *BcMFSR* in growth and morphology was investigated by characterizing Δ*BcmfsR* and Δ*BcmfsR‐com*, along with the WT and Δ*BcmfsR‐T‐DNA*. Both Δ*BcmfsR‐T‐DNA* and Δ*BcmfsR* exhibited a significantly increased number of hyphal tip branches compared to the WT, suggesting altered hyphal growth (Figure 3c,d). Additionally, the hyphal layer thickness was significantly reduced in these mutants, suggesting impaired mycelial development, potentially associated with altered structural integrity or nutrient acquisition (Figure 3c,e). The colony growth rate of both Δ*BcmfsR‐T‐DNA* and Δ*BcmfsR* strains was slightly slower than that of the WT, further supporting the conclusion that the loss of *BcMFSR* impairs normal fungal development and growth (Figure 3c,f). Δ*BcmfsR‐com* restored *B. cinerea* growth characteristics including the number of hyphal tip branches, hyphal layer thickness and colony growth rate, to levels comparable to those observed in the WT. However, the pathogenicity of the three *BcMFSR* mutants did not differ from that of WT (Figure 3g,h). These results indicate that *BcMFSR* plays a role in *B. cinerea* growth and development, but not pathogenicity.

### 2.4 BcMFSR Plays a Critical Role in SIGS

**FIGURE 4:**
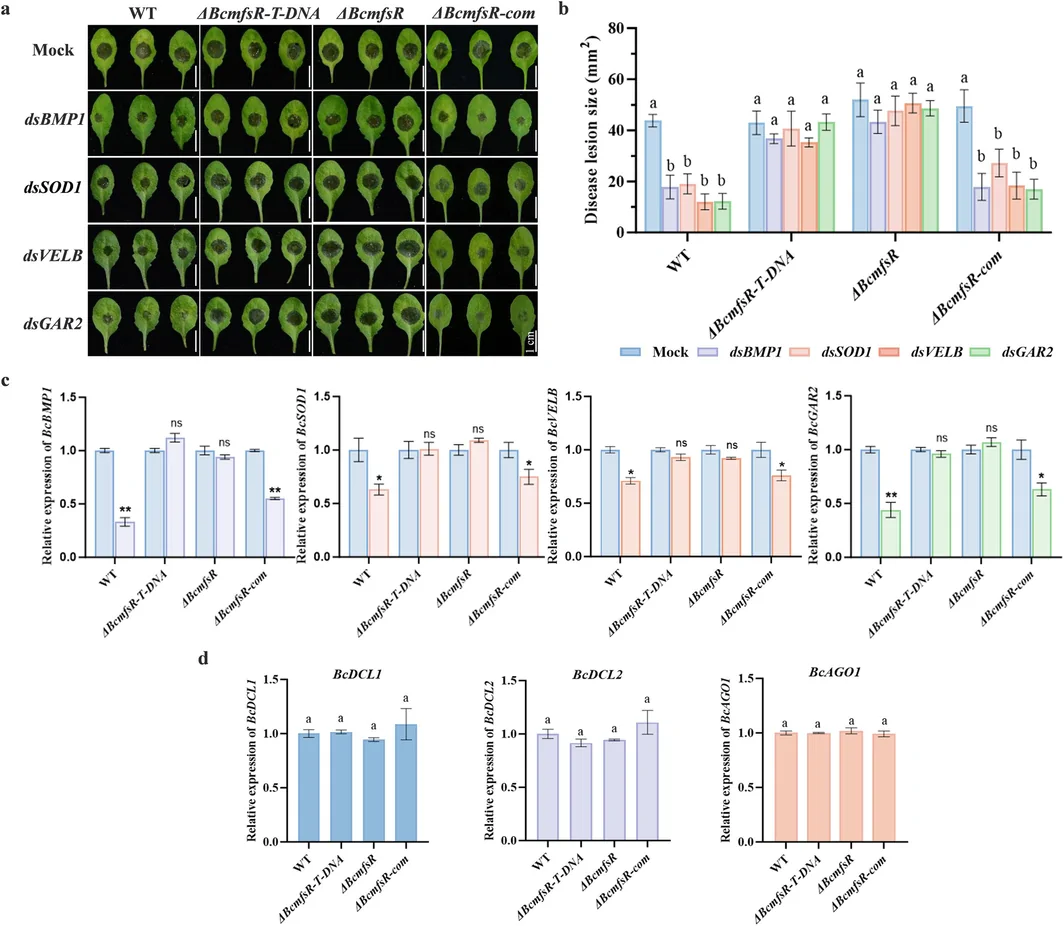
dsRNA fails to reduce the pathogenicity of Δ*BcmfsR*, and the effect is not sequence‐specific. (a) Pathogenicity assay of wild type (WT), Δ*BcmfsR‐T‐DNA*, Δ*BcmfsR* and Δ*BcmfsR‐com* strains on *Arabidopsis thaliana* after external application of *dsGFP*, *dsBMP1*, *dsSOD1*, *dsVELB* and *dsGAR2*. (b) Lesion sizes were measured at 3 days post‐inoculation in *A. thaliana* using ImageJ (c). Reverse transcription‐quantitative PCR (RT‐qPCR) analysis of target gene expression in *Botrytis cinerea*. Statistical analysis used ANOVA and Dunnett's multiple comparison test to identify significant differences; *p < 0.05, **p < 0.01, ns not significant. (d) RT‐qPCR analysis of *BcDCL1*, *BcDCL2* and *BcAGO1* expression in WT, Δ*BcmfsR‐T‐DNA*, Δ*BcmfsR* and Δ*BcmfsR‐com* strains. Statistical analysis was performed using Student's *t*‐test; different lowercase letters indicate significant difference, *p* < 0.05.

Δ*BcmfsR* and Δ*BcmfsR‐com* were then analysed alongside the WT and Δ*BcmfsR‐T‐DNA* (*B. cinerea A10*) to evaluate the function of *BcMFSR* in SIGS and its effect on fungal virulence. Compared to Mock (*dsGFP*) treatment, the Δ*BcmfsR* mutant showed reduced sensitivity to SIGS under *dsBMP1* treatment, producing necrotic lesions similar in size to those caused by the WT (Figure 4a,b). This phenotype resembled that of the Δ*BcmfsR‐T‐DNA* mutant, which was previously described as defective in the SIGS response. In contrast, the Δ*BcmfsR‐com* strain displayed disease symptoms comparable to those of WT, with smaller lesions and reduced hyphal development (Figure 4a,b). This result suggests that the loss of *BcMFSR* affects *dsBMP1*‐mediated SIGS, and this defect is rescued by reintroducing a functional *BcMFSR* gene.

To confirm the effect of *BcMFSR* on gene silencing, we measured *BcBMP1* expression, the target gene of *dsBMP1*‐mediated SIGS. Consistent with the observed disease phenotype, we found that *BcBMP1* expression was significantly reduced in plants infected with the Δ*BcmfsR‐com* strain, similar to that in WT *B. cinerea* (Figure 4c). However, no significant reduction in *BcBMP1* expression was observed in plants infected with Δ*BcmfsR* and Δ*BcmfsR‐T‐DNA*, indicating that the knockout of *BcMFSR* interferes with the gene silencing process caused by SIGS. These results support the hypothesis that *BcMFSR* plays a crucial role in SIGS efficiency in *B. cinerea* , possibly ranging from interrupting dsRNA uptake to generating sRNAs or blocking RNA silencing.

To investigate whether the inhibitory effect on SIGS was general or sequence‐specific, four dsRNAs with different sequences, namely *dsBMP1*, *dsSOD1*, *dsVELB* and *dsGAR2*, were examined (Figure 4a). Pathogenicity assays revealed that neither Δ*BcmfsR‐T‐DNA* nor Δ*BcmfsR* exhibited reduced lesion size after treatment with any of the dsRNAs (Figure 4b). Similarly, no significant differences in the target gene expression level were observed in either Δ*BcmfsR‐T‐DNA* or Δ*BcmfsR* treatment (Figure 4c). In contrast, Δ*BcmfsR‐com* responded similarly to the WT *B. cinerea* , with lesion sizes comparable to those of WT infection and effective silencing of target gene expression observed. These results demonstrate that *BcMFSR* is essential for the silencing efficiency of exogenously applied dsRNA in *B. cinerea* , and is independent of the dsRNA sequence.

To investigate the underlying mechanism by which *BcMFSR* influences SIGS, we analysed the expression of key components of the RNAi machinery in WT, Δ*BcmfsR‐T‐DNA*, Δ*BcmfsR* and Δ*BcmfsR‐com*. The expression of *BcDCL1*, *BcDCL2* and *BcAGO1*, which are involved in small RNA biogenesis and gene silencing, was examined. Surprisingly, no significant differences in gene expression were observed between the mutant and WT strains (Figure 4d). This finding suggests that the disruption of SIGS in Δ*BcmfsR* is not due to a defect in general *B. cinerea* RNAi machinery or small RNA processing in *B. cinerea* . Instead, it points to a more specific biological process upstream of dsRNA cleavage or small RNA‐mediated silencing.

### 2.5 BcMFSR Is Involved in dsRNA Internalization Through Endocytosis

**FIGURE 5:**
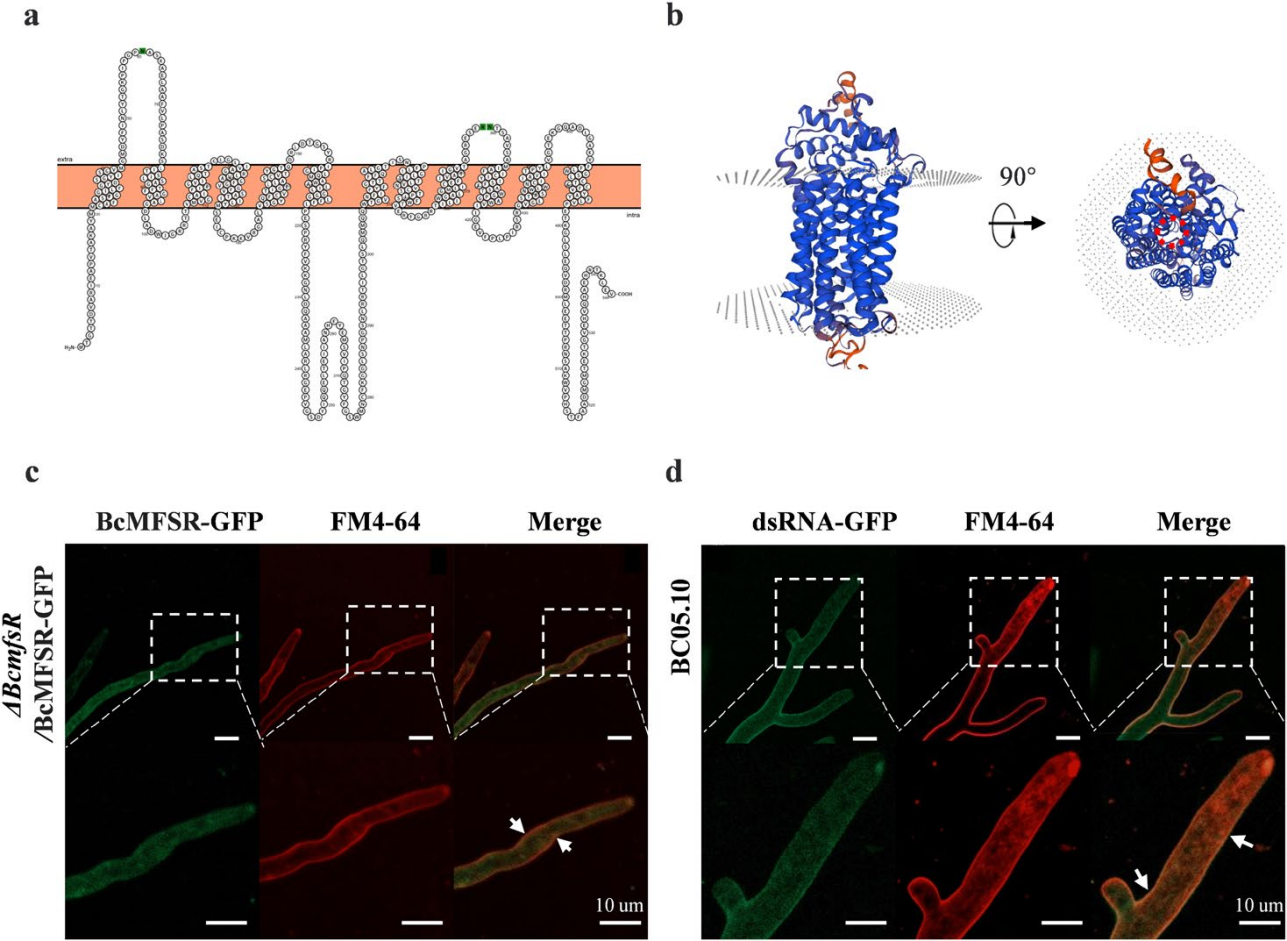
In *Botrytis cinerea*, BcMFSR co‐localizes with vesicles, which also co‐localize with dsRNA. (a) Predicted secondary structure of the BcMFSR protein. The orange region represents the cell membrane. (b) Predicted tertiary structure of the BcMFSR protein. The dotted region represents the cell membrane. The transmembrane helices are shown in the centre, and the structure on the right represents the protein after a 90° rotation. The red dashed circle highlights the cavity formed by the 12 transmembrane helices. (c) Confocal microscopy analysis of the subcellular localization of BcMFSR and FM4‐64‐stained vesicles. (d) Confocal microscopy analysis of the colocalization of FM4‐64‐stained vesicles and fluorescently labelled dsRNA.

Phylogenetic analyses using BcMFSR proteins (XP_001558083.1) and homologues (similarity ≧ 40%) revealed the presence of two highly representative clusters in the neighbour‐joining phylogenetic tree (Figure S4). The first cluster includes BcMFSR and BcDW1, which is a putative monosaccharide transporter, while the second cluster contains closely related proteins. Secondary structure prediction indicated the presence of 12 transmembrane domains typical of membrane‐bound proteins involved in transporting molecules across membranes (Figure 5a). The tertiary structure, visualized as a barrel‐like configuration, suggested a functional role in facilitating cargo transport across the cell membrane (Figure 5b). Previous studies have shown that membrane transport proteins, such as MFSD8, are involved in protein secretion (Huber et al. 2020). To investigate whether BcMFSR follows a similar pathway, a BcMFSR‐GFP fusion was generated in the Δ*BcmfsR* background. BcMFSR‐GFP co‐localized perfectly with vesicular structures labelled by FM4‐64, a fluorescent dye that selectively stains vesicle membranes formed through endocytosis (Figure 5c). These findings indicate that BcMFSR localizes to vesicles integral to dsRNA internalization (Cai et al. 2018). Confocal microscopy revealed that fluorescently labelled dsRNA was internalized into hyphal cells and co‐localized with FM4‐64‐labelled vesicles. Co‐localization was particularly strong at the hyphal tips, where vesicles actively accumulated (Figure 5d). This strong co‐localization between dsRNA and vesicular structures provides compelling evidence that BcMFSR facilitates dsRNA internalization via vesicles, further supporting our hypothesis that BcMFSR plays a critical role in the SIGS process.

**FIGURE 6:**
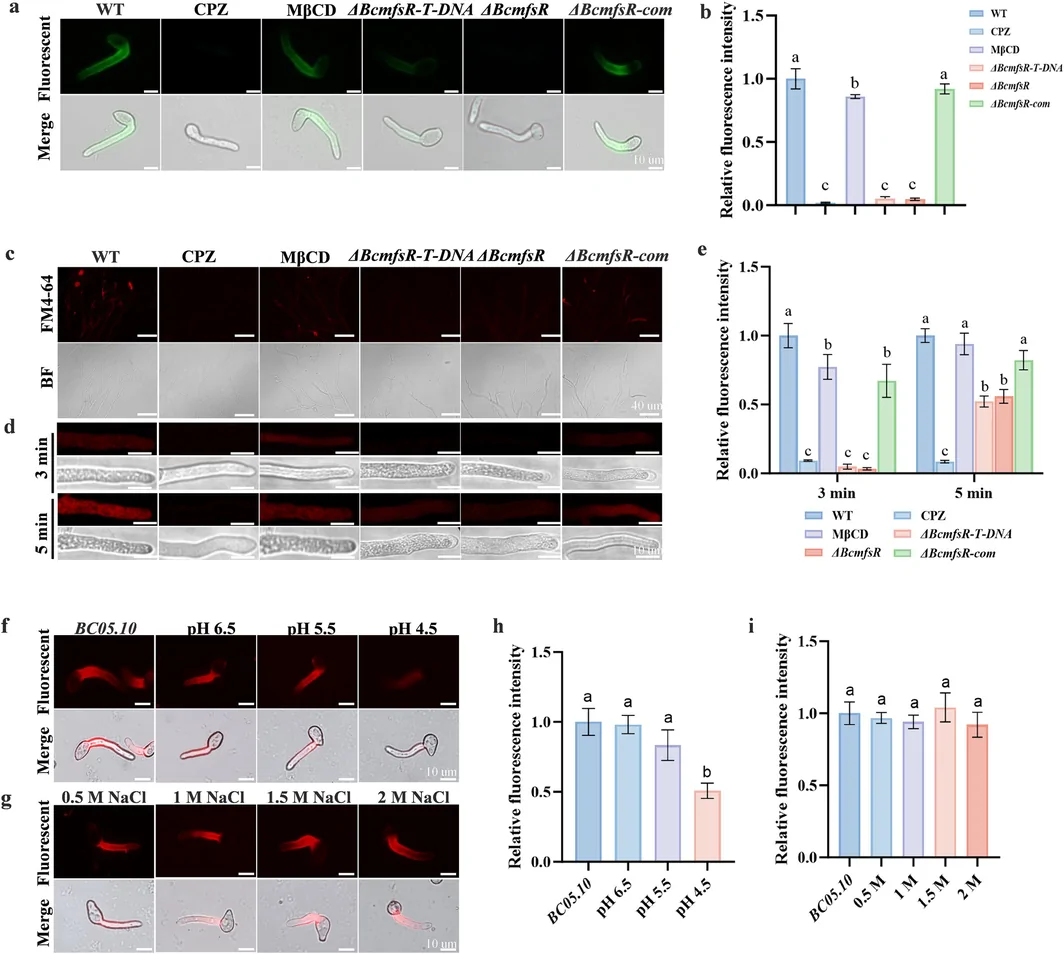
BcMFSR is involved in pH‐dependent dsRNA endocytosis. (a) Wild type (WT, *Botrytis cinerea* BC05.10), Δ*BcmfsR‐T‐DNA*, Δ*BcmfsR*, and Δ*BcmfsR‐com* mutants were incubated with fluorescently labelled dsRNA for 6 h, with chlorpromazine (CPZ) and methyl‐β‐cyclodextrin (MβCD) used as controls. (b) The fluorescence intensity within the hyphae of WT, Δ*BcmfsR‐T‐DNA*, Δ*BcmfsR*, and Δ*BcmfsR‐com* after uptake of fluorescently labelled dsRNA was analysed using ImageJ. (c) WT, Δ*BcmfsR‐T‐DNA*, Δ*BcmfsR*, and Δ*BcmfsR‐com* mutants were incubated with FM4‐64 for 3 min and observed under a fluorescence microscope with a 20× objective. (d) WT, Δ*BcmfsR‐T‐DNA*, Δ*BcmfsR*, and Δ*BcmfsR‐com* mutants were incubated with FM4‐64 for 3 and 5 min. Samples were observed under a laser confocal microscope with a 63× objective. (e) The fluorescence intensity within the hyphae of WT, Δ*BcmfsR‐T‐DNA*, Δ*BcmfsR*, and Δ*BcmfsR‐com* after incubation with FM4‐64 for 3 and 5 min was analysed using ImageJ. (f) The uptake of dsRNA dissolved in acetic acid solutions with varying pH levels by *B. cinerea* was observed with confocal microscopy. (g) The uptake of dsRNA dissolved in NaCl solutions of different concentrations by *B. cinerea* was observed via confocal microscopy. (h, i) Quantification of the relative fluorescence intensity of dsRNA uptake by *B. cinerea* across different pH levels (h) and NaCl concentrations (i).

To determine whether BcMFSR is involved in dsRNA uptake that interrupts *dsBMP1*‐mediated SIGS, dsRNA internalization was assessed in both WT and *BcMFSR* mutants. The endocytosis inhibitors chlorpromazine (CPZ) and methyl‐β‐cyclodextrin (MβCD) were used as controls. CPZ is a cationic amphiphilic compound that inhibits clathrin‐mediated endocytosis by preventing the formation of clathrin‐coated pits at the site of vesicle invagination by translocation of clathrin from the plasma membrane to intracellular vesicles (Vercauteren et al. 2010); MβCD, on the other hand, affects caveolae‐dependent endocytosis (Perez et al. 2011). The results showed that, similar to CPZ‐treated WT, dsRNA uptake was undetected in either the Δ*BcmfsR‐T‐DNA* or Δ*BcmfsR* mutant. In contrast, strong fluorescent signals indicative of successful dsRNA internalization were observed in MβCD‐treated WT and Δ*BcmfsR‐com*, with uptake levels comparable to those of the WT (Figure 6a,b). The loss of fluorescence in the BcMFSR mutants and its restoration in the complemented strain provide genetic evidence that BcMFSR is required for efficient dsRNA uptake in *B. cinerea* . Together with the inhibitory effects of CPZ and MβCD, these findings suggest a potential association between BcMFSR‐mediated dsRNA uptake and clathrin‐mediated endocytosis.

FM4‐64 preferentially associates with the plasma membrane lipid bilayer and is internalized through endocytosis, making it a widely used marker for endocytosis (Fischer‐Parton et al. 2000). We assessed endocytic activity in WT, Δ*BcmfsR‐T‐DNA*, Δ*BcmfsR*, and Δ*BcmfsR‐com* by examining the subcellular distribution of FM4‐64. Fluorescence microscopy revealed that after 3 min of FM4‐64 incubation, red fluorescence signals indicative of dye internalization were observed in WT cells but absent in Δ*BcmfsR‐T‐DNA* or Δ*BcmfsR* mutants (Figure 6c,d). These observations suggest that the FM4‐64 uptake, and by extension, the endocytic process, is severely impaired in both mutant strains. Examination of hyphal tips showed a red fluorescence signal in WT hyphae but not in Δ*BcmfsR‐T‐DNA* and Δ*BcmfsR* mutant hyphae (Figure 6d). After 5 min, a faint fluorescence signal was observable in the mutants, but at a significantly weaker level than in the WT. ImageJ analysis showed that the fluorescence intensity in Δ*BcmfsR‐T‐DNA* and Δ*BcmfsR* was 50%–70% lower than in the WT at both time points (Figure 6e). Together, these results demonstrate that FM4‐64 internalization was less effective in both Δ*BcmfsR‐T‐DNA* and Δ*BcmfsR* mutants, indicating BcMFSR may play a role in the *B. cinerea* endocytosis process, and that it is a vesicle‐related endocytosis, rather than caveolae‐dependent endocytosis.

### 2.6 BcMFSR‐Mediated dsRNA Uptake Is pH‐Dependent

Many membrane‐localized transporters use cross‐membrane gradient to drive cargo transportation (Abramson and Wright 2021). To investigate the effects of pH and NaCl concentration on BcMFSR‐mediated dsRNA uptake, we examined the uptake of fluorescently labelled dsRNA across a pH range (pH 4.5, 5.5, 6.5) and a range of sodium ion concentrations (0.5 to 2 M NaCl). Results showed that lower environmental pH reduced *dsBMP1* uptake (Figure 6f,h). In contrast, varying NaCl concentrations did not affect *dsBMP1* uptake, with no discernible fluorescence differences among samples (Figure 6g,i). To further exclude the possibility that the observed fluorescence changes were caused by pH‐dependent effects on the fluorescent dye, we examined the fluorescence intensity of Cy5‐labelled dsRNA under different pH conditions. Cy5 fluorescence remained stable under the tested pH conditions, indicating that the reduced fluorescence observed under acidic conditions was unlikely to result from fluorophore quenching (Figure S5a). Meanwhile, electrophoretic analysis confirmed that the dsRNAs remained largely intact for at least 6 h under all tested pH and NaCl conditions, suggesting that the reduced fluorescence observed at low pH was not caused by dsRNA degradation (Figure S5b). These results indicate that BcMFSR‐mediated dsRNA uptake is pH‐dependent.

### 2.7 The Full BcMFSR Structure Is Necessary for dsRNA Uptake

**FIGURE 7:**
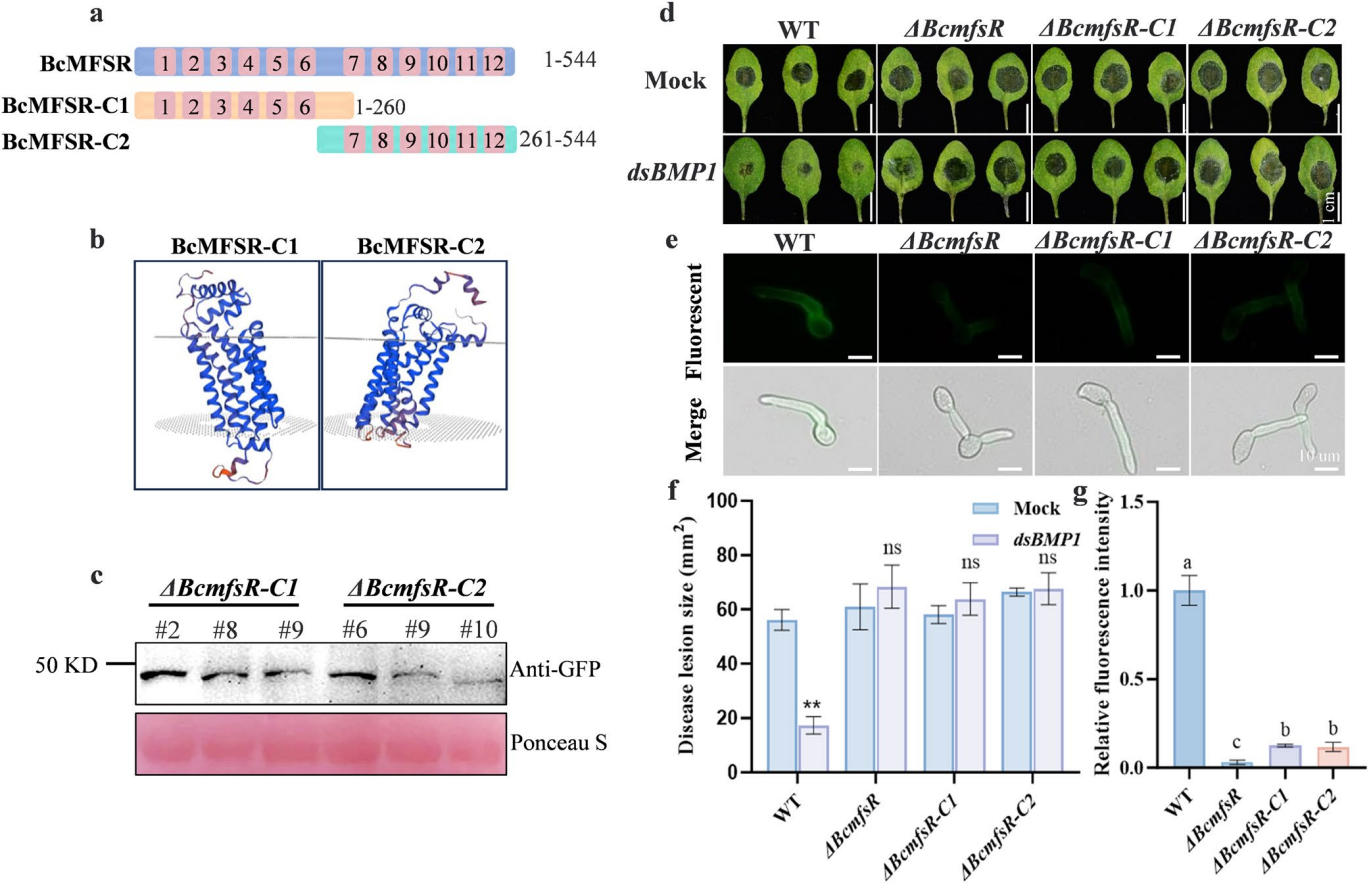
Structural regions of BcMFSR cooperate to mediate uptake of exogenous dsRNA. (a, b) Schematic diagram of BcMFSR domain recovery constructs. (c) Western blot analysis of protein expression in Δ*BcmfsR‐C1* and Δ*BcmfsR‐C2*. Strains are indicated by #; Ponceau S staining was used as the loading control. (d) Pathogenicity assays of wild type (WT), Δ*BcmfsR‐C1* and Δ*BcmfsR‐C2* following exogenous application of dsRNA. (e) Δ*BcmfsR‐C1* and Δ*BcmfsR‐C2* mutants were incubated with fluorescently labelled dsRNA for 6 h. (f) Lesion sizes were measured at 3 days post‐inoculation on *Arabidopsis thaliana* . Significance was determined using Student's *t*‐test: **p < 0.01; ns, not significant. (g) Quantification of fluorescence intensity within the hyphae of WT, Δ*BcmfsR*, Δ*BcmfsR‐C1* and Δ*BcmfsR‐C2* after uptake of fluorescently labelled dsRNA, analysed using ImageJ. Different lowercase letters indicate significant differences (*p* < 0.05).

To investigate the structural functionality of the BcMFSR protein in dsRNA uptake, truncation and complementation mutants were generated based on its structural features. These features include 12 conserved transmembrane α‐helices arranged into two six‐helix bundles (Figure 5c). These helices form an interface cavity between the bundles, which may be oriented towards the cytoplasm or extracellular region depending on the protein's conformational state (Yan 2013). Truncation mutants were generated by deleting specific helices. The study examined Δ*BcmfsR‐C1*, comprising the first six α‐helices (1–6), and Δ*BcmfsR‐C2*, which consists of the last six α‐helices (7–12) (Figure 7a,b). Western blot analysis confirmed successful expression of both Δ*BcmfsR‐C1* and Δ*BcmfsR‐C2*, with the respective protein fragments observed at expected molecular weights (Figure 7c).

To assess the functional implications of these truncation mutants, SIGS assays were performed on WT and mutant strains. Both Δ*BcmfsR‐C1* and Δ*BcmfsR‐C2* displayed disease symptoms identical to the Δ*BcmfsR* strain (Figure 7d), indicating that truncation of either half of the protein abolished BcMFSR‐mediated dsRNA uptake activity. In contrast, Δ*BcmfsR‐com*, which retained all α‐helices, exhibited a disease phenotype similar to that of the WT *B. cinerea* , demonstrating that the full protein structure is necessary for normal function. Statistical analysis revealed that the lesion area of Δ*BcmfsR‐C1* and Δ*BcmfsR‐C2* was similar to that of Δ*BcmfsR*, consistent with the loss of *BcMFSR* function in these truncation mutants (Figure 7d,f). Both truncation mutants exhibited markedly reduced uptake of fluorescently labelled dsRNA (Figure 7e,g) and its internalization ability for FM4‐64 was significantly impaired (Figure S6). Together, these results indicate that an intact full‐length BcMFSR protein is required for dsRNA uptake activity.

## 3 Discussion

RNA‐based disease control strategies have garnered increasing attention for their environmental sustainability, high efficacy, rapid onset of action and non‐transgenic approaches (Wang and Jin 2017). Selecting appropriate target genes is crucial for determining the efficacy of RNA‐based pesticides. Previous studies targeting mitogen‐activated protein kinases (MAPKs) *BMP1* and *BMP3*, both essential for pathogenesis, as well as the tetraspanin protein PLS1 involved in appressorium penetration, have shown significant inhibitory effects against grey mould (Rui and Hahn 2007; Spada et al. 2023).

In this study, we identified three additional target genes in *B. cinerea* : *BcSOD1*, *BcGAR2* and *BcVELB*. These genes participate in distinct biological processes that are important for fungal fitness and pathogenicity. *BcSOD1* contributes to protection against oxidative stress, *BcVELB* acts as a global regulator of fungal growth, development and virulence, whereas *BcGAR2* is involved in d‐galacturonic acid metabolism and pectin utilization. Although the role of *BcGAR2* in early fungal development is less obvious than those of *BcSOD1* and *BcVELB*, perturbation of carbon metabolism may indirectly affect physiological processes required for normal spore germination and subsequent growth. Consistent with the importance of these genes in fungal biology, dsRNAs targeting *BcSOD1*, *BcGAR2* and *BcVELB* effectively suppressed grey mould development on multiple host plants, resulting in significant downregulation of target gene expression and inhibition of spore germination, hyphal growth and infection cushion formation. We therefore established a systematic platform for identifying effective RNAi targets for the control of *B. cinerea* . The inhibitory effects of exogenous dsRNAs were evaluated at multiple developmental stages, providing a useful framework for the selection and validation of target genes for RNA‐based disease control.

### 3.1 Rationale and Validation of a Targeted Mutant Screen for SIGS‐Specific Defect

We designed the genetic screen to identify mutants that are impaired in the SIGS response but retain wild‐type pathogenicity. This design allowed us to separate general virulence factors from genes that specifically control the uptake and processing of exogenous dsRNA. This distinction is paramount, as a mutation that broadly attenuates fungal fitness would artificially mimic a SIGS‐resistant phenotype by reducing lesion formation, irrespective of dsRNA application. Our screening criteria, therefore, were deliberately designed to isolate mutants that lost dsRNA‐mediated protection while remaining fully pathogenic in the absence of dsRNA. The success of this approach is validated by the identification of *BcMFSR*, a mutant that perfectly met these conditions and was subsequently proven to be defective in a fundamental, upstream step of the SIGS pathway: pH‐dependent dsRNA uptake associated with endocytic activity.

The employment of a high‐quality ATMT mutant library was instrumental to this discovery. The validation data—demonstrating high insertion efficiency (93.3%), genomic randomness, and a predominance of single‐copy insertions (86%)—provided critical assurance regarding the library's suitability for unbiased forward genetics. Screening a substantial subset (200 transformants) in triplicate biological assays mitigated experimental noise and established the reproducibility of the observed phenotypes. The subsequent pinpointing of the T‐DNA insertion within the *BcMFSR* locus underscores the precision of this methodology.

### 3.2 BcMFSR Is an Important Factor Associated With dsRNA Uptake and SIGS in *B. cinerea*

Our findings go beyond merely cataloguing a SIGS defect. They reveal a new and unexpected biological role for a member of the widely used Major Facilitator Superfamily. While MFS transporters are known for exporting various substrates, including drugs and toxins, their role in actively importing macromolecules such as dsRNA has not been reported previously. The co‐localization of BcMFSR with endocytic vesicles and the simultaneous loss of both dsRNA and the endocytic tracer (FM4‐64) uptake in the Δ*BcmfsR* mutant strongly suggest that this is essential for a vesicle‐mediated endocytic pathway for dsRNA internalization. These findings suggest a previously unrecognized role for an MFS family protein in dsRNA uptake and provide new insights into the molecular processes associated with SIGS in fungi.

It should be noted that the cellular basis of dsRNA uptake may vary among different developmental stages of the fungus. Although dsRNA treatment significantly delayed spore germination, the uptake experiments in this study were primarily conducted in germ tubes and hyphae, where dsRNA internalization can be more readily visualized. Previous studies have shown that dsRNA uptake is most active at hyphal tips and branching regions, which are sites of active endocytosis (Wytinck et al. 2020). Whether spores employ the same uptake mechanism remains unclear and warrants further investigation.

### 3.3 Potential Role of BcMFSR in pH‐Dependent dsRNA Uptake

Based on the observed effect of extracellular pH on dsRNA uptake, we propose a working hypothesis in which BcMFSR may be functionally associated with the proton electrochemical gradient across the plasma membrane. Plasma membrane H^+^‐ATPases establish and maintain proton gradients that provide the driving force for many secondary transport processes in fungi (Sukhova et al. 2021; Palmgren and Morsomme 2019). In fungi, H^+^‐ATPase activity has been associated with nutrient uptake, pathogenicity, and responses to environmental stress (Yang and Peng 2023; Liu et al. 2023; Mariscal et al. 2022). Thus, BcMFSR‐mediated dsRNA uptake may be influenced by changes in the proton electrochemical gradient established by plasma membrane H^+^‐ATPases. Although members of the MFS superfamily frequently use proton gradients to energize substrate transport, our results only demonstrate that dsRNA uptake is influenced by extracellular pH. Whether proton gradients directly contribute to BcMFSR‐mediated dsRNA uptake remains to be determined.

Despite their functional diversity, many MFS transporters share a conserved structural fold. Their core typically consists of 12 transmembrane helices organized into two structurally similar domains (N‐domain and C‐domain), with the substrate‐binding site located at the interface between the domains (Nigam 2018; Giacomini et al. 2010). The transport mechanism follows the ‘alternate access’ model, in which conformational changes alternately expose the binding site to either side of the membrane (Brandsch 2013), and the ‘rocker‐switch’ model (Daniel et al. 2006; Drew et al. 2021).

These conformational transitions provide a structural basis for substrate translocation in MFS transporters. In some MFS transporters, coordinated binding of substrates and coupling ions can influence the transition between outward‐ and inward‐facing conformations, thereby facilitating secondary transport (Lin et al. 2015). Whether a similar coupling mechanism contributes to BcMFSR‐mediated dsRNA uptake remains an open question. An antiport mechanism involving dsRNA and H^+^ could represent one possible model for how BcMFSR‐mediated uptake may be associated with the proton electrochemical gradient. Future studies could investigate whether BcMFSR undergoes pH‐ or proton gradient‐dependent conformational changes and whether such changes are associated with dsRNA uptake.

In conclusion, our mutant‐library‐based screening approach enabled the identification of BcMFSR as an important factor associated with exogenous dsRNA uptake in *B. cinerea* . This discovery not only identifies BcMFSR as an important component associated with exogenous dsRNA uptake in *B. cinerea* but also opens new avenues for enhancing the efficiency of RNA pesticides by modulating this uptake process or by designing formulations that optimize dsRNA delivery and uptake.

## 4 Experimental Procedures

### 4.1 Plant and Fungal Materials

Wild‐type *A. thaliana* Columbia‐0 (Col‐0) was cultivated in vermiculite. Plants were maintained in a greenhouse at 22°C with 50% humidity, under a 12‐h light and 12‐h dark cycle. *B. cinerea* BC05.10 served as the WT strain for infection assays, constructing the T‐DNA insertion mutant library, gene knockout and complementation, and microscopic studies. Both WT and mutant strains were grown on potato dextrose agar (PDA) with 1.5% agar at room temperature.

### 4.2 Production of dsRNA in *E. coli*

Total RNA was extracted from *B. cinerea* using TRIzol reagent (Thermo Fisher Scientific). Subsequently, cDNA was synthesized with the PrimeScript RT reagent Kit with gDNA Eraser (Perfect Real Time) (TaKaRa). The cDNA from *B. cinerea* served as a template to amplify a 400 bp target gene fragment, which was then digested with SacI and XhoI enzymes (TaKaRa) and cloned into the SacI‐XhoI sites of the L4440 vector. The recombinant L4440 vector containing the target gene fragment was introduced into *E. coli* HT115 (DE3) by transformation. Single colonies were selected and cultured at 37°C with shaking at 220 rpm until the OD_600_ reached 0.5–0.7. dsRNA was induced by adding 0.6 mM isopropyl β‐d‐thiogalactoside (IPTG) and shaking for 5 h. Post‐induction, total RNA was extracted from *E. coli* . For RNase A digestion, 5 μL of dsRNA solution was mixed with 5 μL of 2 M NaCl and 1 μL of RNase A (10 mg/mL). The mixture was incubated at 37°C for 30 min. The integrity and purity of the recovered dsRNA were evaluated by electrophoresis on a 1.3% agarose gel. Density analysis was performed using ImageJ. A 750 bp band from the DL2000 Plus DNA Marker (Vazyme) at 100 ng/5 μL was used as the standard for dsRNA quantification.

### 4.3 RNA Extraction and RT‐qPCR

Infected *B. cinerea* plant samples were collected, and total RNA was extracted with TRIzol reagent (Thermo Fisher Scientific). First‐strand cDNA was synthesized using the PrimeScript RT reagent Kit with gDNA Eraser (Perfect Real Time) (TaKaRa). RT‐qPCR was performed using a Thermo Fisher QuantStudio 5 real‐time PCR system and ChamQ SYBR RT‐qPCR Master Mix (Low ROX Premixed) (Vazyme). The thermal cycling conditions were 95°C for 5 min, followed by 40 cycles of 95°C for 10 s and 60°C for 30 s. Amplification specificity was confirmed by dissociation curve analysis, performed at 95°C for 15 s, 60°C for 1 min, and 95°C for 1 s. Target gene expression was analysed using the same RT‐qPCR protocol as described above, *B. cinerea* actin was used as an internal reference. Relative gene expression was quantified using the 2^−∆∆*C*t^ method.

For RNA extraction from *B. cinerea* , the mycelia were filtered from the culture medium using filter paper, washed three times with double‐distilled water, dried, and ground into a fine powder using liquid nitrogen. Total RNA was isolated with TRIzol reagent following the manufacturer's instructions.

### 4.4 In Vitro Uptake of Fluorescently Labelled dsRNA by *B. cinerea*

T7 promoter sequences were added to both the 5′ and 3′ ends of the RNAi fragment via PCR amplification. The resulting DNA fragments, containing T7 promoters at both ends, were used for in vitro transcription, and fluorescein‐12‐UTR‐labelled dsRNA was synthesized using the High Yield T7 Fluorescein RNA Transcription Kit (Belongbio). Impurities, including free nucleotides and proteins, were removed using a dsRNA purification kit (magnetic bead method) (Vazyme). The uptake of fluorescently labelled dsRNA by *B. cinerea* mycelium was observed using a confocal microscope. A 5 μL volume of fluorescently labelled dsRNA (50 ng/μL) was applied to 5 μL of *B. cinerea* spores at a concentration of 2 × 10^5^ spores/mL. After thorough mixing, the solution was applied to a hydrophobic glass slide and incubated for 6 h. For chemical inhibitor treatment, spores of *B. cinerea* were pre‐incubated with 2 μM CPZ and 2 mM MβCD for 2 h before the addition of dsRNA (Wytinck et al. 2020).

To investigate the effects of pH and NaCl concentration on dsRNA uptake, dsRNA was diluted to a final concentration of 50 ng/μL in acetic acid solutions adjusted to pH 4.5, 5.5 or 6.5, or in NaCl solutions ranging from 0.5 to 2 M. The dsRNA was co‐incubated with *B. cinerea* for 6 h. All fungal growth slides were kept in the dark at 25°C. Germinated spores or mycelium were treated with RNase A to degrade dsRNA present on their surface. This treatment was conducted at 37°C for 30 min, and the fluorescence signal was analysed using an inverted fluorescence microscope.

Cy5‐labelled dsRNA was diluted to a final concentration of 50 ng/μL in buffers at pH 4.5, 5.5 and 6.5 and incubated for 6 h. A volume of 20 μL of each sample was added to each well, and RNase‐free water was used as the blank control. For each pH condition, three technical replicates were included. The fluorescence intensity of Cy5‐labelled dsRNA was measured using a multidetection microplate reader with excitation and emission wavelengths of 640 and 680 nm, respectively. The fluorescence intensity of each sample was normalized to that of the corresponding RNase‐free water control and expressed as relative fluorescence intensity. The experiment was independently repeated three times.

### 4.5 DsRNA Application on Plant Material

A 5 μL aliquot of *B. cinerea* spore suspension (2 × 10^5^ spores/mL) and 20 μL of dsRNA (50 ng/μL) were applied to the surfaces of 4‐week‐old *Arabidopsis*, onion, strawberry and tomato fruits and leaves. For tomato fruit infections, a 5 mm deep wound was made on the surface using a syringe. Infected plant tissues were incubated in a light‐controlled chamber at 22°C for 3 days before analysis. Each treatment included 10 leaves or fruits, and the experiment was repeated three times.

### 4.6 Trypan Blue Staining

A mixture of phenol, glycerol, lactic acid, water and trypan blue was prepared in a 50:50:50:50:0.1 ratio, then diluted with 96% ethanol at a 1:2 ratio. After 48 h of infection, the leaves were incubated in a diluted trypan blue solution and then boiled for 1 min. The leaves were then left at room temperature overnight. The dye solution was removed, and the leaves were placed in a 2.5 g/mL chloral hydrate solution for decolourization, immersion in 70% glycerol for microscopic observation. Three leaves were treated per experiment, and the experiment was repeated three times.

### 4.7 In Vitro Spore Germination Assay

A 5 μL volume of *B. cinerea* spore suspension (2 × 10^5^ spores/mL) and 5 μL of dsRNA (50 ng/μL) were applied to a hydrophobic glass slide and incubated in the dark for 3–48 h. The samples were then observed under a 20× microscope, and six photos were taken for each treatment. The experiment was repeated three times. Three types were set up for separate statistical analysis: germination, delayed germination, and inhibited germination.

The uptake of fluorescently labelled dsRNA by the mycelia of *B. cinerea* WT and mutant strains was observed using an inverted fluorescence microscope. The method for treating *B. cinerea* spores with dsRNA is as described above, and six photos were taken for each treatment. The experiment was repeated three times.

### 4.8 Construction of a *B. cinerea* Mutant Library via the ATMT Method and Validation of the Library

A *B. cinerea* T‐DNA tagged mutant library, consisting of approximately 3000 transformants, was generated in the WT strain BC05.10 using the ATMT method as previously described (Schumacher et al. 2014). The mutant library was constructed using the binary vector pKHT, which contains a hygromycin resistance cassette that includes the *A. nidulans* *trpC* promoter and the hygromycin phosphotransferase gene (*HPH*). The pKHT plasmid was then transformed into *A. tumefaciens* EHA105 for expression. The *Agrobacterium* harbouring the recombinant plasmid was streaked onto Luria Bertani (LB) agar plates supplemented with 50 μg/mL kanamycin and 50 μg/mL rifampicin. A single colony was selected and cultured in LB liquid medium with the same antibiotics at 28°C and 200 rpm for 24 h. The *Agrobacterium* culture was transferred into minimal medium (MM) broth containing 50 μg/mL kanamycin and incubated at 28°C and 200 rpm for 48 h. The culture was then diluted with induction medium (IM) to an OD_600_ of 0.15 and further incubated at 28°C and 200 rpm for an additional 6 h. The *Agrobacterium* cell suspension was mixed with the collected conidia to reach a final spore concentration of 10^5^ spores/mL. Solid CO‐IM medium plates were prepared, and the mixture of conidia and *Agrobacterium* was applied to the surface of the plates. Co‐cultivation was performed at 22°C for 48 h. After co‐cultivation, the plates were overlaid with PDA supplemented with 50 μg/mL hygromycin and 500 μg/mL cephalosporin. Emerging colonies were transferred to fresh PDA plates containing 50 μg/mL hygromycin for further growth. Colonies that grew under these conditions were considered transformants. The transformants were subcultured three times on PDA supplemented with 50 μg/mL hygromycin to confirm stability.

Colonies that continued to grow were considered stable and stored at −80°C for long‐term preservation.

To assess the insertional randomness of the mutant library was evaluated using the RAPD method (Xena de Enrech 2000). A pool of 20 random primers was used for the analysis. Total DNA from various insertion mutants was amplified by PCR, and polymorphic bands were generated by gel electrophoresis and analysed.

The copy number of the exogenous genes in the transformants was quantified using qPCR, following the methods of Gadaleta et al. (2011) and Yang et al. (2005) with minor modifications. Initially, the pKHT plasmid was used as the standard for the exogenous *HPH* gene to establish the standard curve, with *B. cinerea* *actin* serving as the internal reference gene and the wild‐type *B. cinerea* as the negative control. The standard curve was generated using a linear regression model. Subsequently, the Pfaffl method was used to estimate the copy number of the exogenous gene in the transformant. The amplification efficiency (*E*) of the target gene was calculated from the slope (*r*) of the standard curve equation. The control sample used was a verified single‐copy transgenic material. The ratio of the exogenous gene to the reference gene was determined by calculating Δ*C*_t_ and adjusting for the amplification efficiency, which was then used to estimate the exogenous gene copy number. *E* = 10^(−1/*r*)^ – 1, N = (*E*_*HPH*_^Δ*C*t (control − sample)^)/(*E*_*ACT*_^Δ*C*t (control − sample)^), where *r* is the slope of the standard curve; *E* is the amplification efficiency; N is the estimated copy number.

### 4.9 Screening of *B. cinerea* Mutant Library

Individual transformants were selected and cultured on PDA supplemented with 50 μg/mL hygromycin for 2 days to promote growth. Fresh mycelial plugs were excised from the colony edges and transferred onto fresh PDA containing hygromycin for further propagation. After 7 days of incubation at 25°C, the transformed spore suspension was harvested. *dsGFP* was used as the negative control for dsRNA treatment, while WT *B. cinerea* served as the positive control after dsRNA application. A total of 5 μL of transformed spore suspension (2 × 10^5^ spores/mL) and 20 μL of dsRNA (50 ng/μL) were applied to the surfaces of *Arabidopsis* leaves. Ten leaves were used for each treatment. Photos were taken, and the treated leaves were examined after 3 days of incubation. The experiment was repeated three times.

### 4.10 HiTAIL‐PCR to Verify the Flanking Sequences at the T‐DNA Insertion Sites

HiTAIL‐PCR was used to amplify the sequences flanking the T‐DNA insertions in the mutants. The synthesis of left‐ and right‐border nested primers and random primers, as well as the PCR programme, was designed following the method described by Liu and Chen (2007). Primer sequences are provided in Table S1. The amplified flanking sequences were compared with the *B. cinerea* genome database (http://fungi.ensembl.org/Botrytis_cinerea/Info/Index) to accurately determine the T‐DNA insertion sites.

### 4.11 Secondary and Tertiary Structure Prediction of BcMFSR

The Protter server (http://wlab.ethz.ch/protter/start/) was used to illustrate the secondary structure of the *B. cinerea* MFS‐encoded protein. The Phyre2 server (http://www.sbg.bio.ic.ac.uk/phyre2) was used to automate homology modelling of this protein's tertiary structure (Kelley et al. 2015).

### 4.12 Phylogenetic Analysis of BcMFSR Protein

Homologous BcMFSR proteins were identified by BLASTP searches against the NCBI database using the BcMFSR amino acid sequence as the query. Proteins sharing at least 40% sequence identity with BcMFSR and an *E*‐value < 1e−5 were selected for phylogenetic analysis. Amino acid sequences were aligned using ClustalW implemented in MEGA 7. A neighbour‐joining phylogenetic tree was constructed in MEGA 7 using the Poisson correction model, and branch support was evaluated with 1000 bootstrap replicates. Positions with less than 50% site coverage were eliminated.

### 4.13 Construction of *BcMFSR* Knockout and Complementation Strains

To construct the knockout vector, 1 kb upstream and downstream sequences flanking the *BcMFSR* gene were retrieved from the *B. cinerea* genome database (http://fungi.ensembl.org/Botrytis_cinerea/Info/Index) to serve as the left and right homologous arms. These arms were then recombined with the *HPH* gene, which is approximately 1.7 kb in size and contains the *trpC* promoter from *A. nidulans* . The ClonExpress Ultra One Step Cloning Kit V2 (Vazyme) was used to ligate the recombinant fragment into the pMD‐19 T vector via homologous recombination. After sequence validation, the gene knockout vector was successfully assembled.

To construct the complementation vector, the *BcMFSR* promoter was first predicted using the online tool (http://www.fruitfly.org/seq_tools/promoter.html), and a 2 kb fragment comprising the promoter region and open reading frame (ORF) (excluding the stop codon) was amplified. The amplified fragment was ligated into the XhoI‐digested pYF11‐neo plasmid, which contains the *GFP* gene sequence, using homologous recombination (ClonExpress Ultra One Step Cloning Kit V2, Vazyme). Following sequence validation, the recombinant vector carrying the GFP marker was successfully constructed.

The regional complementation vectors were constructed by splitting BcMFSR into two structural regions, designated as C1 and C2, based on its predicted secondary structure. The schematic diagram of vector construction is presented in the manuscript, and the methodology follows that of the previously described vector. All primer sequences are provided in Table S1.

### 4.14 Polyethylene Glycol‐Mediated Protoplast Transformation

The polyethylene glycol (PEG)‐mediated protoplast transformation method for *B. cinerea* was performed with minor modifications, as previously described by Hamada et al. (1994). Fresh *B. cinerea* mycelium was harvested and treated with 20 mL of 0.5% Glucanex (Sigma) per gram in KCl buffer. The protoplasts were filtered through a 25 μm nylon membrane and centrifuged at 1200 *g* for 10 min. The protoplasts were washed twice with ice‐cold KCl buffer, followed by a single wash with 1 mL of STC solution, and then resuspended in 800 μL of STC solution. Subsequently, 200 μL of SPTC solution was added dropwise. A total of 5 μg of target DNA and 5 μL of spermidine (50 mM storage solution) were added to 100 μL of protoplast suspension (10^8^ cells/mL). After gentle mixing, the suspension was incubated on ice for 30 min. Next, 1 mL of SPTC solution was added, gently mixed, and incubated at room temperature for 20 min. Then, 10–20 mL of regeneration medium was added, and the culture was incubated overnight at 25°C with shaking at 100 rpm. The recovered mycelium was carefully transferred into 200 mL regeneration medium prewarmed to 43°C, then plated onto a culture dish. After 24 h of incubation at 25°C, the culture was overlaid with selective medium containing 50 μg/mL hygromycin. After 5 days of incubation, transformants were selected and subcultured on fresh PDA supplemented with 50 μg/mL hygromycin. The transformant mycelium was passaged three times on PDA supplemented with 50 μg/mL hygromycin to ensure stability.

### 4.15 FM4‐64 Staining

Fresh *B. cinerea* mycelium, precultured on PDA plates for 48 h, was then inoculated onto glass slides coated with a thin layer of PDA and incubated with 6 μM FM4‐64 for various time intervals, starting 24 h after culture. The excitation wavelength for FM4‐64 was set to 559 nm, and fluorescence signals were captured and analysed using Zeiss confocal microscopy at 20× and 63× magnification. At least six independent microscopic fields were examined for each treatment in three independent biological replicates.

### 4.16 Western Blotting

Total protein extraction was performed using 5× SDS‐PAGE loading buffer (250 mM Tris–HCl, pH 6.8, 10% SDS, 0.5% bromophenol blue, 50% glycerol, and 5% β‐mercaptoethanol). Protein detection was carried out using the SuperPico ECL Chemiluminescence Kit (Vazyme) and an anti‐GFP antibody (Abmart). Ponceau S staining was used as a loading control for total protein quantification.

### 4.17 Statistical Analysis

All statistical analyses were performed using GraphPad Prism (v. 10.0.2). Each experiment included at least three independent biological replicates, and data are presented as mean ± SE. For comparisons across multiple treatment groups, one‐way ANOVA was used, followed by Tukey's post hoc test (or the least significant difference method) for pairwise comparisons (*p* < 0.05). Student's *t*‐tests were used to compare two groups. Quantitative data, including lesion area, disease fluorescence intensity, and greyscale values, were analysed with ImageJ software (v. 1.54) and plotted using GraphPad Prism. Prior to statistical analysis, data distributions and variance homogeneity were examined, and no obvious deviations from normality or homoscedasticity were observed.

## Author Contributions

**Le Xu:** writing – original draft, data curation, methodology, software, investigation, formal analysis. **Ze Yu:** software, data curation, validation, writing – review and editing. **Ruonan Du:** validation, data curation, visualization. **Huijie Ma:** validation. **Xiaoxiao Hu:** validation. **Ran Wei:** validation. **Qiwang Hu:** validation. **Cong Sheng:** validation. **Wambui Doris Njoki:** writing – review and editing. **Pan Chen:** validation. **Xuechao Shi:** validation. **Cheng Tang:** validation. **Huameng Zhang:** validation, supervision. **Shaoxia Zhou:** validation, supervision. **Haoming Zhong:** validation. **Weishu Wang:** validation. **Isashova Umida:** writing – review and editing. **Hongwei Zhao:** writing – review and editing, writing – original draft, resources, funding acquisition, project administration, conceptualization, supervision.

## Funding

This work was supported by the Yangtze River Delta Science and Technology Innovation Consortium Key Research Project (Grant No. 24CSJ140200) and the National Natural Science Foundation of China (Grant No. 32372556) to Hongwei Zhao.

## Conflicts of Interest

The authors declare no conflicts of interest.

## Supporting information


**Figure S1:** dsRNA enters *Botrytis cinerea* and affects the formation of infection cushion. (a) Representative images of hyphal development (12 h) and infection cushion formation (24 h) in *B. cinerea* treated with different dsRNAs. (b) Stability assay of dsRNA in *B. cinerea* spores 48 h after treatment. (c) Detection of fluorescently labelled dsRNA uptake by wild‐type *B. cinerea* . (d) Reverse transcription‐quantitative PCR analysis of target gene expression in *B. cinerea* treated with *dsBMP1*.

**Figure S2:** Supplementary analyses of the *Botrytis cinerea* mutant library and screening procedure. (a) Quantitative PCR analysis for quantification of copy numbers in *B. cinerea* T‐DNA insertion mutants. (b) Screening flowchart for the *B. cinerea* mutant library.

**Figure S3:** Screening of the *Botrytis cinerea* T‐DNA insertion mutant library. (a) Pathogenicity screening of *B. cinerea* T‐DNA mutants after exogenous application of dsRNA. (b) Lesion size quantification of *B. cinerea* T‐DNA mutants following treatment with *dsGFP* and *dsBMP1*. (c) HiTAIL‐PCR was used to identify the sequences flanking the T‐DNA insertion site in the *B32* and *B36* transformants. LB‐2b and RB‐2b represent left and right border nested primers, respectively, while LAD1–4 represent four long degenerate primers. Potential target bands are circled in red. (d) Schematic representation of the T‐DNA insertion sites in the *B32* and *B36* transformants within the *B. cinerea* genome. Purple squares indicate the 5′ untranslated region (UTR), blue squares represent exons, grey lines depict introns, yellow squares indicate the 3′ UTR, and black squares represent T‐DNA.

**Figure S4:** Phylogenetic tree of the BcMFSR protein constructed using the neighbour‐joining method.

**Figure S5:** dsRNA stability and fluorescence intensity of Cy5‐labelled dsRNA are not affected by pH or NaCl conditions. (a) Relative fluorescence intensity of Cy5‐labelled dsRNA under different pH conditions. (b) RNA integrity of Cy5‐labelled dsRNA under different pH and NaCl conditions.

**Figure S6:** Reduced endocytic activity in the Δ*BcmfsR‐C1* and Δ*BcmfsR‐C2* mutants.

**Table S1:** Primers used in this research.

## Data Availability

The gene sequences used in this study are available in *EnsemblFungi* under the following accession numbers: *BcBMP1* (*Bcin02g08170*), *BcSOD1* (*Bcin03g03390*), *BcVELB* (*Bcin01g02730*), *BcGAR2* (*Bcin03g01500*), *BcMFSR* (*Bcin12g02300*), *BcDCL1* (*Bcin12g06230*), *BcDCL2* (*Bcin14g03910*), and *BcAGO1* (*Bcin02g09270*).

## References

[mpp70343-bib-0001] Abramson, J. , and E. M. Wright . 2021. “Function Trumps Form in Two Sugar Symporters, *LacY* and *vSGLT* .” International Journal of Molecular Sciences 22: 3572. 10.3390/ijms22073572 PMC8037263 33808202

[mpp70343-bib-0002] Brandsch, M. 2013. “Drug Transport via the Intestinal Peptide Transporter PepT1.” Current Opinion in Pharmacology 13: 881–887. 10.1016/j.coph.2013.08.004 24007794

[mpp70343-bib-0003] Cai, Q. , L. Qiao , M. Wang , et al. 2018. “Plants Send Small RNAs in Extracellular Vesicles to Fungal Pathogen to Silence Virulence Genes.” Science 360: 1126–1129. 10.1126/science.aar4142 PMC6442475 29773668

[mpp70343-bib-0004] Chen, P. , Y. Lan , S. Ding , et al. 2025. “RNA Interference‐Based dsRNA Application Confers Prolonged Protection Against Rice Blast and Viral Diseases, Offering a Scalable Solution for Enhanced Crop Disease Management.” Journal of Integrative Plant Biology 67: 1633–1648. 10.1111/jipb.13896 40226962

[mpp70343-bib-0005] Chen, X. , and O. Rechavi . 2022. “Plant and Animal Small RNA Communications Between Cells and Organisms.” Nature Reviews Molecular Cell Biology 23: 185–203. 10.1038/s41580-021-00425-y PMC9208737 34707241

[mpp70343-bib-0006] Daniel, H. , B. Spanier , G. Kottra , and D. Weitz . 2006. “From Bacteria to Man: Archaic Proton‐Dependent Peptide Transporters at Work.” Physiology 21: 93–102. 10.1152/physiol.00054.2005 16565475

[mpp70343-bib-0007] Drew, D. , R. A. North , K. Nagarathinam , and M. Tanabe . 2021. “Structures and General Transport Mechanisms by the Major Facilitator Superfamily (MFS).” Chemical Reviews 121: 5289–5335. 10.1021/acs.chemrev.0c00983 PMC8154325 33886296

[mpp70343-bib-0008] Duanis‐Assaf, D. , O. Galsurker , O. Davydov , et al. 2022. “Double‐Stranded RNA Targeting Fungal Ergosterol Biosynthesis Pathway Controls *Botrytis cinerea* and Postharvest Grey Mould.” Plant Biotechnology Journal 20: 226–237. 10.1111/pbi.13708 PMC8710829 34520611

[mpp70343-bib-0009] Fischer‐Parton, S. , R. M. Parton , P. C. Hickey , J. Dijksterhuis , H. A. Atkinson , and N. D. Read . 2000. “Confocal Microscopy of FM4‐64 as a Tool for Analysing Endocytosis and Vesicle Trafficking in Living Fungal Hyphae.” Journal of Microscopy 198: 246–259. 10.1046/j.1365-2818.2000.00708.x 10849201

[mpp70343-bib-0010] Gadaleta, A. , A. Giancaspro , M. F. Cardone , and A. Blanco . 2011. “Real‐Time PCR for the Detection of Precise Transgene Copy Number in Durum Wheat.” Cellular & Molecular Biology Letters 16: 652–668. 10.2478/s11658-011-0029-5 PMC6275630 21922222

[mpp70343-bib-0011] Giacomini, K. M. , S. M. Huang , D. J. Tweedie , et al. 2010. “Membrane Transporters in Drug Development.” Nature Reviews Drug Discovery 9: 215–236. 10.1038/nrd3028 PMC3326076 20190787

[mpp70343-bib-0012] Hamada, W. , P. Reignault , G. Bompeix , and M. Boccara . 1994. “Transformation of *Botrytis cinerea* With the Hygromycin‐B Resistance Gene, *Hph* .” Current Genetics 26: 251–255. 10.1007/BF00309556 7859308

[mpp70343-bib-0013] He, B. , H. Wang , G. Liu , et al. 2023. “Fungal Small RNAs Ride in Extracellular Vesicles to Enter Plant Cells Through Clathrin‐Mediated Endocytosis.” Nature Communications 14: 243–254. 10.1038/s41467-023-40093-4 PMC10359353 37474601

[mpp70343-bib-0014] Huber, R. J. , S. Mathavarajah , and S. Q. Yap . 2020. “Mfsd8 Localizes to Endocytic Compartments and Influences the Secretion of Cln5 and Cathepsin D in *Dictyostelium* .” Cellular Signalling 70: 1095–1099. 10.1016/j.cellsig.2020.109572 32087303

[mpp70343-bib-0052] Kelley, L. A. , S. Mezulis , C. M. Yates , M. N. Wass , and M. J. Sternberg . 2015. “The Phyre2 Web Portal for Protein Modeling, Prediction and Analysis.” Nature Protocols 10: 845–858. 10.1038/nprot.2015.053 PMC5298202 25950237

[mpp70343-bib-0015] Koch, A. , D. Biedenkopf , A. Furch , et al. 2016. “An RNAi‐Based Control of *Fusarium graminearum* Infections Through Spraying of Long Dsrnas Involves a Plant Passage and Is Controlled by the Fungal Silencing Machinery.” PLoS Pathogens 12: e1005901. 10.1371/journal.ppat.1005901 PMC5063301 27737019

[mpp70343-bib-0016] Li, H. , Y. Chen , Z. Q. Zhang , B. Q. Li , G. Z. Qin , and S. P. Tian . 2018. “Pathogenic Mechanisms and Control Strategies of *Botrytis cinerea* Causing Post‐Harvest Decay in Fruits and Vegetables.” Food Quality and Safety 2: 111–119.

[mpp70343-bib-0017] Lin, L. , S. W. Yee , R. B. Kim , and K. M. Giacomini . 2015. “SLC Transporters as Therapeutic Targets: Emerging Opportunities.” Nature Reviews Drug Discovery 14: 543–560. 10.1038/nrd4626 PMC4698371 26111766

[mpp70343-bib-0018] Liu, Y. , and V. A. Bankaitis . 2010. “Phosphoinositide Phosphatases in Cell Biology and Disease.” Progress in Lipid Research 49: 201–217. 10.1016/j.plipres.2009.12.001 PMC2873057 20043944

[mpp70343-bib-0019] Liu, Y. , Y. T. Xi , Y. Y. Lv , et al. 2023. “The Plasma Membrane H^+^ ATPase CsPMA2 Regulates Lipid Droplet Formation, Appressorial Development and Virulence in *Colletotrichum siamense* .” International Journal of Molecular Sciences 24. 10.3390/ijms242417337 PMC10743824 38139168

[mpp70343-bib-0020] Liu, Y. G. , and Y. Chen . 2007. “High‐Efficiency Thermal Asymmetric Interlaced PCR for Amplification of Unknown Flanking Sequences.” BioTechniques 43: 649–654. 10.2144/000112601 18072594

[mpp70343-bib-0021] Luo, X. , S. Nanda , Y. Zhang , X. Zhou , C. Yang , and H. Pan . 2024. “Risk Assessment of RNAi‐Based Biopesticides.” New Crops 1: 100019.

[mpp70343-bib-0022] Marger, M. D. , and M. H. Saier . 1993. “A Major Superfamily of Transmembrane Facilitators That Catalyse Uniport, Symport and Antiport.” Trends in Biochemical Sciences 18: 13–20. 10.1016/0968-0004(93)90081-w 8438231

[mpp70343-bib-0023] Mariscal, M. , C. Miguel‐Rojas , C. Hera , T. R. Fernandes , and A. Di Pietro . 2022. “ *Fusarium oxysporum* Casein Kinase 1, a Negative Regulator of the Plasma Membrane H^+^‐ATPase Pma1, Is Required for Development and Pathogenicity.” Journal of Fungi 8: 1300. 10.3390/jof8121300 PMC9786551 36547634

[mpp70343-bib-0024] Nigam, S. K. 2018. “The SLC22 Transporter Family: A Paradigm for the Impact of Drug Transporters on Metabolic Pathways, Signaling, and Disease.” Annual Review of Pharmacology and Toxicology 58: 663–687. 10.1146/annurev-pharmtox-010617-052713 PMC6225997 29309257

[mpp70343-bib-0025] Ouyang, S. Q. , H. M. Ji , T. Feng , S. J. Luo , L. Cheng , and N. Wang . 2023. “Artificial Trans‐Kingdom RNAi of *FolRDR1* Is a Potential Strategy to Control Tomato Wilt Disease.” PLoS Pathogens 19: e1011463. 10.1371/journal.ppat.1011463 PMC10313012 37339156

[mpp70343-bib-0026] Palmgren, M. , and P. Morsomme . 2019. “The Plasma Membrane H^+^‐ATPase, a Simple Polypeptide With a Long History.” Yeast 36: 201–210. 10.1002/yea.3365 PMC6590192 30447028

[mpp70343-bib-0027] Perez, A. P. , M. L. Cosaka , E. L. Romero , and M. J. Morilla . 2011. “Uptake and Intracellular Traffic of siRNA Dendriplexes in Glioblastoma Cells and Macrophages.” International Journal of Nanomedicine 6: 2715–2728. 10.2147/IJN.S25235 PMC3218585 22114502

[mpp70343-bib-0028] Qiao, L. , C. Lan , L. Capriotti , et al. 2021. “Spray‐Induced Gene Silencing for Disease Control Is Dependent on the Efficiency of Pathogen RNA Uptake.” Plant Biotechnology Journal 19: 1756–1768. 10.1111/pbi.13589 PMC8428832 33774895

[mpp70343-bib-0029] Reddy, V. S. , M. A. Shlykov , R. Castillo , E. I. Sun , and M. H. Saier Jr. 2012. “The Major Facilitator Superfamily (MFS) Revisited.” FEBS Journal 279: 2022–2035. 10.1111/j.1742-4658.2012.08588.x PMC3425384 22458847

[mpp70343-bib-0030] Rolke, Y. , S. J. Liu , T. Quidde , et al. 2004. “Functional Analysis of H_2_O_2_‐Generating Systems in *Botrytis cinerea*: The Major Cu‐Zn‐Superoxide Dismutase (*BCSOD1*) Contributes to Virulence on French Bean, Whereas a Glucose Oxidase (*BCGOD1*) is Dispensable.” Molecular Plant Pathology 5: 17–27. 10.1111/j.1364-3703.2004.00201.x 20565578

[mpp70343-bib-0031] Rui, O. , and M. Hahn . 2007. “The Slt2‐Type MAP Kinase *Bmp3* of *Botrytis cinerea* Is Required for Normal Saprotrophic Growth, Conidiation, Plant Surface Sensing and Host Tissue Colonization.” Molecular Plant Pathology 8: 173–184. 10.1111/j.1364-3703.2007.00383.x 20507489

[mpp70343-bib-0032] Schumacher, J. , A. Simon , K. C. Cohrs , M. Viaud , and P. Tudzynski . 2014. “The Transcription Factor *BcLTF1* Regulates Virulence and Light Responses in the Necrotrophic Plant Pathogen *Botrytis cinerea* .” PLoS Genetics 10: e1004040. 10.1371/journal.pgen.1004040 PMC3886904 24415947

[mpp70343-bib-0033] Song, P. , C. Q. Cai , M. Skokut , B. D. Kosegi , and J. F. Petolino . 2002. “Quantitative Real‐Time PCR as a Screening Tool for Estimating Transgene Copy Number in WHISKERS‐Derived Transgenic Maize.” Plant Cell Reports 20: 948–954.

[mpp70343-bib-0034] Spada, M. , C. Pugliesi , M. Fambrini , D. Palpacelli , and S. Pecchia . 2023. “Knockdown of *Bmp1* and *Pls1* Virulence Genes by Exogenous Application of RNAi‐Inducing dsRNA in *Botrytis cinerea* .” International Journal of Molecular Sciences 24: 342–354. 10.3390/ijms24054869 PMC10003348 36902297

[mpp70343-bib-0035] Sukhova, E. , D. Ratnitsyna , and V. Sukhov . 2021. “Stochastic Spatial Heterogeneity in Activities of H^+^‐ATP‐Ases in Electrically Connected Plant Cells Decreases Threshold for Cooling‐Induced Electrical Responses.” International Journal of Molecular Sciences 22: 8254. 10.3390/ijms22158254 PMC8348073 34361018

[mpp70343-bib-0036] Veloso, J. , and A. L. van Kan . 2018. “Many Shades of Grey in *Botrytis*–Host Plant Interactions.” Trends in Plant Science 23: 613–622. 10.1016/j.tplants.2018.03.016 29724660

[mpp70343-bib-0037] Vercauteren, D. , R. E. Vandenbroucke , A. T. Jones , et al. 2010. “The Use of Inhibitors to Study Endocytic Pathways of Gene Carriers: Optimization and Pitfalls.” Molecular Therapy 18: 561–569. 10.1038/mt.2009.281 PMC2839427 20010917

[mpp70343-bib-0038] Wang, M. , and H. Jin . 2017. “Spray‐Induced Gene Silencing: A Powerful Innovative Strategy for Crop Protection.” Trends in Microbiology 25: 4–6. 10.1016/j.tim.2016.11.011 PMC5182084 27923542

[mpp70343-bib-0039] Wang, M. , A. Weiberg , F. M. Lin , B. P. Thomma , H. D. Huang , and H. Jin . 2016. “Bidirectional Cross‐Kingdom RNAi and Fungal Uptake of External RNAs Confer Plant Protection.” Nature Plants 2: 151–161. 10.1038/nplants.2016.151 PMC5040644 27643635

[mpp70343-bib-0040] Weiberg, A. , M. Wang , F. M. Lin , et al. 2013. “Fungal Small RNAs Suppress Plant Immunity by Hijacking Host RNA Interference Pathways.” Science 342: 118–123. 10.1126/science.1239705 PMC4096153 24092744

[mpp70343-bib-0041] Wytinck, N. , D. S. Sullivan , K. T. Biggar , et al. 2020. “Clathrin Mediated Endocytosis Is Involved in the Uptake of Exogenous Double‐Stranded RNA in the White Mold Phytopathogen *Sclerotinia sclerotiorum* .” Scientific Reports 10: 127–139. 10.1038/s41598-020-69771-9 PMC7391711 32728195

[mpp70343-bib-0042] Xena de Enrech, N. 2000. “A Decade of the RAPD Method: Possibilities and Limitations for Plant Genetics Relationship Studies.” Acta Científica Venezolana 51: 197–206. 11460789

[mpp70343-bib-0043] Yan, N. 2013. “Structural Advances for the Major Facilitator Superfamily (MFS) Transporters.” Trends in Biochemical Sciences 38: 151–159. 10.1016/j.tibs.2013.01.003 23403214

[mpp70343-bib-0044] Yang, L. T. , J. Y. Ding , C. M. Zhang , et al. 2005. “Estimating the Copy Number of Transgenes in Transformed Rice by Real‐Time Quantitative PCR.” Plant Cell Reports 23: 759–763. 10.1007/s00299-004-0881-0 15459795

[mpp70343-bib-0045] Yang, Q. Q. , Y. F. Chen , and Z. H. Ma . 2013. “Involvement of *BcVeA* and *BcVelB* in Regulating Conidiation, Pigmentation and Virulence in *Botrytis cinerea* .” Fungal Genetics and Biology 50: 63–71. 10.1016/j.fgb.2012.10.003 23147398

[mpp70343-bib-0046] Yang, S. Z. , and L. T. Peng . 2023. “Significance of the Plasma Membrane H^+^‐ATPase and V‐ATPase for Growth and Pathogenicity in Pathogenic Fungi.” Advances in Applied Microbiology 124: 31–53. 10.1016/bs.aambs.2023.07.001 37597947

[mpp70343-bib-0047] Zhang, L. , and J. A. van Kan . 2013. “ *Botrytis cinerea* Mutants Deficient in D‐Galacturonic Acid Catabolism Have a Perturbed Virulence on *Nicotiana benthamiana* and *Arabidopsis*, but Not on Tomato.” Molecular Plant Pathology 14: 19–29. 10.1111/j.1364-3703.2012.00825.x PMC6638916 22937823

[mpp70343-bib-0048] Zhao, J. H. , and H. S. Guo . 2022. “RNA Silencing: From Discovery and Elucidation to Application and Perspectives.” Journal of Integrative Plant Biology 64: 476–498. 10.1111/jipb.13213 34964265

[mpp70343-bib-0049] Zhao, J. H. , Q. Y. Liu , Z. M. Xie , and H. S. Guo . 2024. “Exploring the Challenges of RNAi‐Based Strategies for Crop Protection.” Advanced Biotechnology 2: 23. 10.1007/s44307-024-00031-x PMC11740845 39883232

[mpp70343-bib-0050] Zheng, L. , M. Campbell , J. Murphy , S. Lam , and J. R. Xu . 2000. “The *BMP1* Gene Is Essential for Pathogenicity in the Gray Mold Fungus *Botrytis cinerea* .” Molecular Plant–Microbe Interactions 13: 724–732. 10.1094/MPMI.2000.13.7.724 10875333

[mpp70343-bib-0051] Zhu, C. , J. H. Liu , J. H. Zhao , et al. 2022. “A Fungal Effector Suppresses the Nuclear Export of AGO1‐miRNA Complex to Promote Infection in Plants.” Proceedings of the National Academy of Sciences of the United States of America 119: e2114583119. 10.1073/pnas.2114583119 PMC8944911 35290117

