# Supplementary material for: BcMFSR Is Required for Efficient Uptake of Exogenous dsRNA and Spray‐Induced Gene Silencing in Botrytis cinerea
Source: Mol Plant Pathol. 2026 Sep 20;27(9):e70343. doi: 10.1111/mpp.70343 (PMC13591111; doi:10.1111/mpp.70343)
Supplement: Supplementary file 2 [file MPP-27-e70343-s002.docx]

**Figure S3. Screening of the *B. cinerea* T-DNA insertion mutant library.**

**(a)** Pathogenicity screening of *B. cinerea* T-DNA mutants after exogenous application of dsRNA. **(b)** Lesion size quantification of *B. cinerea* T-DNA mutants following treatment with *dsGFP* and *dsBMP1*. **(c)** HiTAIL-PCR was used to identify the sequences flanking the T-DNA insertion site in the *B32* and *B36* transformants. LB-2b and RB-2b represent left and right border nested primers, respectively, while LAD1–4 represent four long degenerate primers. Potential target bands are circled in red. **(d)** Schematic representation of the T-DNA insertion sites in the *B32* and *B36* transformants within the *B. cinerea* genome. Purple squares indicate the 5′UTR, blue squares represent exons, gray lines depict introns, yellow squares indicate the 3′UTR, and black squares represent T-DNA.
