# Supplementary material for: BcMFSR Is Required for Efficient Uptake of Exogenous dsRNA and Spray‐Induced Gene Silencing in Botrytis cinerea
Source: Mol Plant Pathol. 2026 Sep 20;27(9):e70343. doi: 10.1111/mpp.70343 (PMC13591111; doi:10.1111/mpp.70343)
Supplement: Supplementary file 3 [file MPP-27-e70343-s003.docx]

| Table S1: Primers used in this research. | |
| --- | --- |
| Primer | Sequences |
| *BcVELB*-F | CGAGCTCTATGGCTGCGTCCATAGGTCC |
| *BcVELB*-R | CCTCGAGAGGGAAAATGAAATATCCAGCTTCAT |
| *BcGAR2*-F | CGAGCTCATGGCTTCAGATTTGAAACCCCTC |
| *BcGAR2*-R | CCTCGAGGGATTTTACGTTCAATAGCGTAGAGAG |
| *BcSOD1*-F | CCTCGAGATGGTTAAAGCAGTTGCTACCGT |
| *BcSOD1*-R | CGAGCTCGCTTCGTTCTCTCCCTTACC |
| *BcBMP1*-F | CGAGCTCGATGACAGCTCGTGCGCCTAAT |
| *BcBMP1*-R | CCTCGAGAGGCTCGCAGGGTTTGATAGATAAAGT |
| *GFP*-F | CGAGCTCGCAACTTGTCTGGTGTCAAAAATAATA |
| *GFP*-R | CCTCGAGGCGCCTGATGCGGTATTTT |
| pKHT-HPH-F | GGGAGCTGTTGGCTGGC |
| pKHT-HPH-R | GGGGAGAGGCGGTTTGC |
| HPH-qF | GGTGTCGTCCATCACAGTTT |
| HPH-qR | CCGCAAGGAATCGGTCAATA |
| BcActin-qF | ACTCATATGTTGGAGATGAAGCGCA |
| BcActin-qR | AATGTTACCATACAAATCCTTACGGA |
| SBS A01 | CAGGCCCTTC |
| SBS A02 | TGCCGAGCTG |
| SBS A03 | AGTCAGCCAC |
| SBS A04 | AATCGGGCTG |
| SBS A05 | AGGGGTCTTG |
| SBS A06 | GGTCCCTGAC |
| SBS A07 | GAAACGGGTG |
| SBS A08 | GTGACGTAGG |
| SBS A09 | GGGTAACGCC |
| SBS A10 | GTGATCGCAG |
| SBS B01 | GTTTCGCTCC |
| SBS B02 | TGATCCCTGG |
| SBS B03 | CATCCCCCTG |
| SBS B04 | GGACTGGAGT |
| SBS B05 | TGCGCCCTTC |
| SBS B06 | TGCTCTGCCC |
| SBS B07 | GGTGACGCAG |
| SBS B08 | GTCCACACGG |
| SBS B09 | TGGGGGACTC |
| SBS B10 | CTGCTGGGAC |
| LAD1-1 | ACGATGGACTCCAGAGCGGCCGCVNVNNNGGAA |
| LAD1-2 | ACGATGGACTCCAGAGCGGCCGCBNBNNNGGTT |
| LAD1-3 | ACGATGGACTCCAGAGCGGCCGCVVNVNNNCCAC |
| LAD1-4 | ACGATGGACTCCAGAGCGGCCGCBDNBNNNCGGT |
| AC | ACGATGGACTCCAGAG |
| RB-0b | CGTGACTGGGAAAACCCTGGCGTT |
| RB-1b | ACGATGGACTCCAGTCCGGCCCAACTTAATCGCCTTGCAGCACATC |
| RB-2b | GAAGAGGCCCGCACCGATCGCCCTT |
| LB-0b | GGGTTCCTATAGGGTTTCGCTCATG |
| LB-1b | CATGTGTTGAGCATATAAGAAACCCT |
| LB-2b | GAATTAATTCGGCGTTAATTCAGT |
| Bcin12g02300-qF | TGCTCCACTCCAATCTCTTTC |
| Bcin12g02300-qR | CAACGATGAACTCGCAGATTAAC |
| Bcin10g04070-qF | CCGCTGTCTCAACTCTCAAT |
| Bcin10g04070-qR | GATACCAGATGCTGGATACCTTC |
| GFP-T7-F | TAATACGACTCACTATAGGGAGAATGGTGAGCAAGGGCG |
| GFP-T7-R | TAATACGACTCACTATAGGGAGACCTCCTTGAAGTCGATGCC |
| BcMFSR-up-F | CCCCCTAAACTCTAATCCCCAT |
| BcMFSR-up-R | AAATTGATGAATTTGATTTTTGTGATT |
| BcMFSR-HPH-F | CAAATTCATCAATTTCTATTCCTTTGCCCTCGGA |
| BcMFSR-HPH-R | GGCCATAGCTGATGAAAAAGCCTGAAC |
| BcMFSR-down-F | CAGCTATGGCCCAGCCG |
| BcMFSR-down-R | TGTTTGCCCCAGTCGTTAGG |
| BcMFSR-mcherry-F | AGCATCGATCCCGGGGTCGACATGACTGGAACCACGGATGTCG |
| BcMFSR-mcherry-R | GCCCTTGCTCACCATGGTACCAACCTCGAGCTTTGTATTATGCTCA |
| BcMFSR-C1-F | ACTCACTATAGGGCGAATTGGGTACTCAAATTGGTTTTTTAAGCTGGTACACCCGA |
| BcMFSR-C1-R | CACCACCCCGGTGAACAGCTCCTCGCCCTTGCTCACGATGGTACGACGACCGAAAC |
| BcMFSR-C2-F | CGGACTAACTCCTCGGCTGGGCCATAGCTATGAAAGTTCGTGGTGCTTTAGTTTC |
| BcMFSR-C2-R | CACCACCCCGGTGAACAGCTCCTCGCCCTTGCTCACAACCTCGAGCTTTGTATTATGC |
| BcMFSR-C-F | ACTCACTATAGGGCGAATTGGGTACTCAAATTGGTTTTTTAAGCTGGTACACCCGA |
| BcMFSR-C-R | CACCACCCCGGTGAACAGCTCCTCGCCCTTGCTCACAACCTCGAGCTTTGTATTATGC |
| Promoter-F | TTTTAAGCTGGTACACCCG |
| Promoter-R | AGCTATGGCCCAGCCGA |
| BcAGO1-qF | GCTTCTTCGCTCGTGGTATT |
| BcAGO1-qR | CACGAATTACAGGACCCTTCTC |
| BcDCL1-qF | AAGAACGAGGCCACAGAATTTA |
| BcDCL1-qR | GTGAGTCAACGAAGGCAGATAG |
| BcDCL2-qF | CGGTAAACTAGAATCGCCCTAC |
| BcDCL2-qR | GTGCATCTGGAAAGTCTGGATA |
