# Supplementary material for: BcMFSR Is Required for Efficient Uptake of Exogenous dsRNA and Spray‐Induced Gene Silencing in Botrytis cinerea
Source: Mol Plant Pathol. 2026 Sep 20;27(9):e70343. doi: 10.1111/mpp.70343 (PMC13591111; doi:10.1111/mpp.70343)
Supplement: Supplementary file 4 [file MPP-27-e70343-s004.docx]

**Figure S5. dsRNA stability and fluorescence intensity of Cy5-labeled dsRNA are not affected by pH or NaCl conditions.**

**(a)** Relative fluorescence intensity of Cy5-labeled dsRNA under different pH conditions. **(b)** RNA integrity of Cy5-labeled dsRNA under different pH and NaCl conditions.
