# Supplementary material for: BcMFSR Is Required for Efficient Uptake of Exogenous dsRNA and Spray‐Induced Gene Silencing in Botrytis cinerea
Source: Mol Plant Pathol. 2026 Sep 20;27(9):e70343. doi: 10.1111/mpp.70343 (PMC13591111; doi:10.1111/mpp.70343)
Supplement: Supplementary file 5 [file MPP-27-e70343-s005.docx]

**Figure S2. Supplementary analyses of the *B. cinerea* mutant library and screening procedure.**

**(a)** qPCR analysis for quantification of copy numbers in *B. cinerea* T-DNA insertion mutants. **(b)** Screening flowchart for the *B. cinerea* mutant library
