# Supplementary material for: BcMFSR Is Required for Efficient Uptake of Exogenous dsRNA and Spray‐Induced Gene Silencing in Botrytis cinerea
Source: Mol Plant Pathol. 2026 Sep 20;27(9):e70343. doi: 10.1111/mpp.70343 (PMC13591111; doi:10.1111/mpp.70343)
Supplement: Supplementary file 6 [file MPP-27-e70343-s006.docx]

**Figure S4. Phylogenetic tree of the BcMFSR protein constructed using the neighbor-joining method.**
