# Supplementary material for: BcMFSR Is Required for Efficient Uptake of Exogenous dsRNA and Spray‐Induced Gene Silencing in Botrytis cinerea
Source: Mol Plant Pathol. 2026 Sep 20;27(9):e70343. doi: 10.1111/mpp.70343 (PMC13591111; doi:10.1111/mpp.70343)
Supplement: Supplementary file 7 [file MPP-27-e70343-s007.docx]

**Figure S1. dsRNA enters *B. cinerea* and affects the formation of infection cushion.**

**(a)** Representative images of hyphal development (12 h) and infection cushion formation (24 h) in *B. cinerea* treated with different dsRNAs. **(b)** Stability assay of dsRNA in *B. cinerea* spores 48 hours after treatment. **(c)** Detection of fluorescently labeled dsRNA uptake by wild-type *B. cinerea*. **(d)** qRT-PCR analysis of target gene expression in *B. cinerea* treated with *dsBMP1*.
